# Motivations, benefits, and barriers to the use of HIV pre-exposure prophylaxis based on the health belief model*

**DOI:** 10.1590/1518-8345.7357.4653

**Published:** 2025-11-10

**Authors:** Rita de Cássia Pereira Rodrigues, Marcela Antonini, Laís do Espirito Santo Lima, Daniel de Macedo Rocha, Elucir Gir, Renata Karina Reis

**Affiliations:** 1 Universidade de São Paulo, Escola de Enfermagem de Ribeirão Preto, PAHO/WHO Collaborating Centre for Nursing Research Development, Ribeirão Preto, SP, Brazil. Universidade de São Paulo Escola de Enfermagem de Ribeirão Preto PAHO/WHO Collaborating Centre for Nursing Research Development SP Ribeirão Preto Brazil

**Keywords:** HIV, Pre-Exposure Prophylaxis, Disease Prevention, Health Belief Model, Nursing, Sexually Transmitted Diseases

## Abstract

to understand the motivations, benefits, and barriers to the use of HIV pre-exposure prophylaxis.

a qualitative study grounded on the Health Belief Model. Sixteen people using HIV pre-exposure prophylaxis were followed up at two Testing and Counseling Centers in a large municipality in the interior of the state of São Paulo, Brazil. Data were collected through individual audio-recorded interviews. Data analysis followed Rosenstock’s Health Belief Model and Bardin’s Content Analysis.

susceptibility to HIV was influenced by uncertainty about partners’ serology and condom failure. The severity of HIV is perceived as greater due to its incurability. Benefits include greater protection, safety, and well-being. Barriers involve difficulties in access, side effects, and social stigma.

the study revealed that the motivation for using Pre-Exposure Prophylaxis was the perception of susceptibility to HIV, the severity of the disease, and benefits such as protection and psychological well-being, while barriers involved difficulties in access, complexity of the clinical protocol, and stigma. Knowing the beliefs and barriers can favor more effective interventions by the health team.

## Highlights

**(1)** Understanding the beliefs of Pre-Exposure Prophylaxis users to support the development of effective interventions. **(2)** Compiles evidence in support of combined HIV prevention strategies. **(3)** Reflects the challenges and barriers faced by Pre-Exposure Prophylaxis users. **(4)** Highlights the need for strategies focused on health information.

## Introduction

Despite scientific, diagnostic, and therapeutic advances, infection with the Human Immunodeficiency Virus (HIV) still represents a contemporary challenge, and its epidemiological, social, cultural, economic, and health impacts are widely documented in the literature^(1)^. Considering the magnitude of the problem, the United Nations General Assembly proposed in the Agenda for Sustainable Development to control the epidemic by 2030 through public information and awareness about different prevention strategies, such as the use of Pre-Exposure Prophylaxis (PrEP)^(2)^.

PrEP is a highly effective biomedical HIV prevention intervention that represents an essential component of the combined prevention recommended by the World Health Organization (WHO) and implemented in several countries since 2015^(3)^. In Brazil, it was incorporated into the Unified Health System (UHS) in 2017, targeting sexually active adults and adolescents at increased risk of infection^(4)^. Since its implementation, 222,948 people have adhered to the strategy. Despite the growth in the number of users, the discontinuation rate is high, reflecting the challenges and barriers to the use and continuity of PrEP^(5)^.

This combined prevention strategy involves continuous care that includes raising awareness among people at substantial risk of acquiring HIV, regular testing, recruitment, adherence, and retention in care^(6)^. However, gaps in this care modality are evident, suggesting the need for further research to understand the determinants, associated factors, and predictors of adherence, as well as the barriers and challenges in implementing the strategy and the disproportionate burden of infection evident among vulnerable groups^(7-9)^.

Therefore, understanding the reasons for using PrEP, its benefits, and the potential barriers faced in using this strategy among people exposed to HIV, is essential for developing effective interventions to improve the care provided to this population. Thus, this study aimed to understand the motivations, benefits, and barriers to using PrEP for HIV.

## Method

### Study design

This is a descriptive study with a qualitative approach conducted in accordance with the guidelines proposed by the Consolidated Criteria for Reporting Qualitative Research (COREQ)^(10)^ and based on the Health Belief Model (HBM)^(11)^.

### Theoretical framework

The HBM was developed in 1966 to explain and understand human behavior in the health process and in the control of diseases such as HIV^(12-13)^. It is a widely referenced model applied in research on health and nursing behaviors. According to the HBM, for a person to act preventively, they need to believe that: a) they are susceptible to a disease or undesirable condition; b) its occurrence is severe for some aspect of their life; c) performing a prophylactic intervention is effective in reducing the susceptibility or severity of the disease (benefits); and d) preventive action does not involve many barriers^(11)^.

A person’s beliefs about these factors determine their likelihood of engaging in health-promoting behavior. They are used to explain the adoption of behaviors that can aid in the prevention and control of diseases such as HIV, as well as to identify the acceptance of recommendations on health care^(12-13)^.

Perceived susceptibility refers to the perception of subjective risks of contracting a condition. Perceived severity refers to a person’s judgment regarding the implications of a diagnosis on their life, such as their work, family life, or social relationships. Benefits are positive actions and preventive measures related to the consequences of the diagnosis. Barriers are related to negative aspects, such as obstacles, discomfort, and financial expenses, among others^(11)^.

### Data collection location

The study settings were two Testing and Counseling Centers (TCCs) in a large municipality in the state of São Paulo, Brazil. It should be noted that TCCs are strategic units for counseling and early diagnosis of Sexually Transmitted Infections (STIs/HIV), and access to these services is conditional on prior scheduling and consultation with an interdisciplinary team for testing, guidance, and counseling.

It is worth mentioning that the municipality studied was one of the pioneers in the country to implement PrEP in its healthcare network and has a total of five TCCs, which are distributed across five health districts. Thus, we chose to select the two TCCs that were the first locations to provide PrEP care and dispensing in the municipality in question, one TCC (TCC-A) located in a region that is easily accessible to the population seeking care for PrEP use, which has people using PrEP in follow-up for a longer period of time; and the other TCC (TCC-B) located in a region of the municipality that serves people living in areas with greater socioeconomic vulnerability. Therefore, we sought to include PrEP users with different sociodemographic characteristics.

### Period

The study was conducted between February and May 2023.

### Population and selection criteria

In the municipality studied, PrEP was implemented in December 2017, and by 2023, 1,623 people had used PrEP, of whom 1,199 had at least one dispensation from the UHS in the last 12 months^(4)^. HIV-negative individuals over the age of 18 who had been using PrEP for at least 90 days and were being monitored by the health service participated in the study. New cases of PrEP were excluded because possible adherence difficulties are considered to be incipient during the early stages of prophylaxis. Sampling was based on intentional convenience criteria, i.e., participants who were present on the collection days and who met the established inclusion criteria were selected.

### Participants

Sixteen participants were invited to participate in the study and there were no dropouts or refusals, with 13 being treated at TCC-A and 3 at TCC-B. The selection of participants sought to include men and women using PrEP from different age groups, aiming to include people with different experiences, perceptions, and motivations.

### Data collection

The data were collected through individual interviews and audio-recorded by the principal investigator, who was trained for this study. The script consisted of open-ended questions based on the HBM that addressed aspects related to the use of PrEP. A pilot study was conducted with four participants to analyze the feasibility, adequacy, and revision of the questionnaire, as well as to allow the researcher to become familiar with the study settings and participants. It should be noted that the participants in the pilot study were not included in the study, and the estimated average time for the interviews was 15 minutes.

After the pilot, the script with questions related to the experience of using PrEP was adjusted by the study researchers in conjunction with the research group with expertise in the subject, being adapted to its final version that included the characterization of the participants, as well as the following guiding questions: Perceived susceptibility: What motivated you to seek PrEP?; Perceived severity: Why did you start using PrEP? What factors influenced your adherence to PrEP?; Perceived benefits: What do you think are the benefits of using PrEP?; Perceived barriers: What do you think are the barriers/disadvantages of using PrEP? To protect data confidentiality, participants were identified with the letter “P” designating “Participant” in the order of inclusion (P1, P2 to P16).

Data collection was carried out with the proper authorization from the municipal Health Department. The research was approved by the Research Ethics Committee (REC) and agreed upon with those responsible for the TCCs. A private, quiet room was made available for the interviews, which took place weekly, from 8 a.m. to 5 p.m., and were conducted by the principal investigator on the days when PrEP users were scheduled to be seen by the health teams.

Participants who were at the TCC for a follow-up consultation regarding PrEP use and who possibly met the inclusion criteria defined in the study were approached individually by the principal investigator, who introduced herself and explained the study objectives in a private room before or after the medical consultation. After the participants gave their written consent by signing the Free and Informed Consent Term (FICT), the researcher conducted individual interviews, with the help of a script and a voice recorder.

### Data analysis and processing

All interviews were transcribed in full, and data collection was carried out until data saturation was achieved, as discussed among the authors^(14)^. Data analysis was guided by Rosenstock’s HBM^(11)^. Content analysis was performed as proposed by Bardin^(15)^, considering the following steps for data organization: pre-analysis, exploration of the material, and interpretation, with treatment by coding. Coding and validation were performed by the authors themselves, without the use of software, respecting the “code tree” of themes grouped into thematic meanings for the subsequent generation of categories. It should be noted that the categories were previously defined based on the theoretical model adopted in the study.

### Ethical aspects

The project was approved by the Health Secretariat of the municipality studied and by the REC of the responsible institution, under opinion No. 4,852,588, dated July 16, 2021. All participants were informed about the objectives, benefits, and risks of the study, with subsequent signing of the informed consent form.

## Results

The sample consisted of 16 participants, with a mean age of 36.5 years. Most were male, representing 15 (93.75%) participants, of whom 13 (81.25%) were Men Who Have Sex With Men (MSM) and 10 (62.50%) were single. Regarding the type of partnership, five (31.25%) had a steady partner, while 10 (62.50%) had casual partners. In terms of education, nine (56.25%) had completed high school, three (18.75%) had a bachelor’s degree, one (6.25%) had a postgraduate degree, and another (6.25%) had a technical degree.

**Figura 1-:**
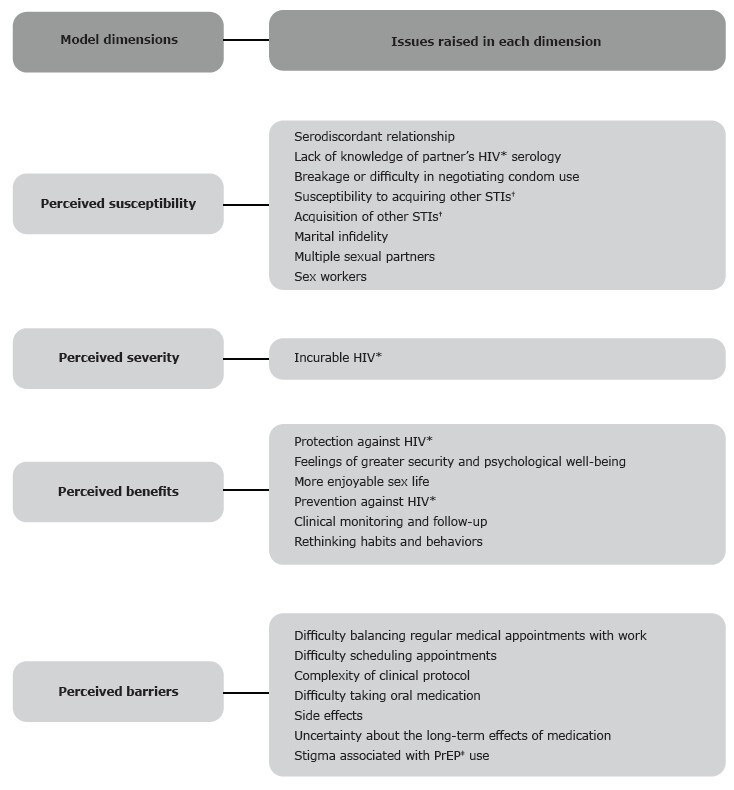
Emerging themes according to each dimension of the Health Belief Model. Ribeirão Preto, SP, Brazil, 2023

The categories resulting from the analysis process respected the dimensions of the HBM and were expressed by 22 themes. When reflecting on the use of PrEP for HIV, participants recognized that although treatment exists, there is no cure. They also recognized the benefits of medication and clinical follow-up. However, difficulties are present, related to accessibility to health services, stigma, side effects, and uncertainties about the repercussions of the medication. The dimensions of the proposed model, as well as the themes that make up each domain, are described in Figura 1.

*HIV = Human Immunodeficiency Virus; ^†^STIs = Sexually Transmitted Infections; ^‡^PrEP = Pre-Exposure Prophylaxis

### Perceived susceptibility

People’s accurate perception of their susceptibility to HIV infection motivated their decision to seek PrEP. Several factors were identified, such as being in a relationship with someone living with HIV, or not knowing/being uncertain about their sexual partner’s HIV status.

*Because in her case* [my partner] *has AIDS, she has HIV, she doesn’t have the disease, just the virus, and “then” it’s a preventive measure since she has it, she gets treated, and I have to do it with her.* (P1)

*First of all, I learned about it because of my previous relationship, because of his HIV, so when I became single, I continued taking it because I thought it was more feasible to protect myself, in addition to condoms, and now with my new serodiscordant relationship, what motivates me is the issue of prevention itself, I think it’s better to take this extra precaution.* (P15)

*It wasn’t just because of this relationship, but for others, no one comes with a sign on their forehead saying they have HIV, and not everyone has the freedom to talk about it.* (P6)

For participants, the use of PrEP provides greater safety and freedom against exposure and potential acquisition of HIV, so its use was also motivated by the perception of the limitations of condoms as a method of prevention due to situations involving accidents such as breakage, or even by resistance and difficulty in negotiating condom use or dislike of using them with their partner.

[...] *and not everyone has the freedom to speak up, so if an accident were to happen, I would use other methods, but I would be more exposed.* (P12)

*Well, actually, we feel a little awkward talking about it, but even when using condoms, there was always, how can I put it, a feeling of mistrust, fear, a feeling that I could also have contracted HIV, for example. And then there’s also the issue of not liking sex with a condom, you know?* (P8)

*To feel safer, to feel protected, because he [my partner] can’t have sex with a condom, and I was feeling very exposed.* (P13)

Susceptibility to HIV infection has also been described in the uncertainty of relationships, even among stable partners, due to the lack of guarantee regarding the other person’s behavior, as well as in specific situations after experiencing other STIs or marital infidelity.

*Because I think it’s extra protection in case the condom breaks, and I even thought that if I had a steady partner, you never know if you’re going to be cheated on or not, so at least when it comes to HIV, I know I’d be okay.* (P12)

*What motivated me was that I already had an STI, and the first time I went for a test, I panicked. Then I thought that I would have to take care of myself, that I needed to be monitored and take better care of myself.* (P14)

*My husband cheated on me, so I realized that you can’t trust anyone.* (P4)

Perceived susceptibility to HIV acquisition was also identified among those who reported multiple sexual partners or were sex workers.

*Because I don’t have a steady partner, I’m very exposed, and so the chances of contracting the virus are also very high, which is why I started taking PrEP to protect myself from the virus.* (P7)

*When we get into prostitution, we do it to make money, to earn a living, to support ourselves. For me, it’s a really cool, normal profession, because through prostitution I met a lot of good people and got what I wanted. I was really afraid of catching the disease, and in prostitution, there are prices for services, right? And there are services that are much more lucrative than others, and I didn’t do them, so I always ended up falling behind, very limited. And then after PrEP, I succeeded.* (P15)

### Perceived severity

For those interviewed, HIV infection can cause damage to their health, as the disease is treatable but incurable. In contrast, there is a perception of lower risk or severity in the case of contracting other STIs.

*So, I went more because of her, because I didn’t know that prevention existed. I knew that treatment existed for HIV, and since she had to be treated, I looked for where “then” I came to be treated, and since we were partners, they said that I had to undergo treatment too, because there is no cure yet, so it will last for the rest of our lives.* (P1)

*There are others* [referring to other STIs]*, of course, everything goes back to square one, right? I know I’m protected if you think about hepatitis, which has vaccines and stuff. But it’s much bigger [referring to HIV] and I’m much more afraid.* (P1)

### Perceived benefits

The benefits identified with the use of PrEP were a feeling of greater protection against HIV and autonomy, resulting in feelings of greater security and psychological well-being. Feeling safe allowed people to enjoy their sex lives more pleasurably.

*I think it’s liberating, it gives me extra reassurance, an extra opportunity in life.* (P5)

*Reassurance, right? From combined prevention, from having another means of preventing HIV.* (P16)

*[...] the main benefit is that you don’t have to worry about the possibility of becoming infected [...].* (P3)

*Having a little more confidence, right? Because accidents happen, so using PrEP makes me a little more confident about the risk of getting infected.* (P12)

*The benefit is also that I feel more at ease, I don’t have that worry of coming here and having a positive result waiting for me. So in that regard, it helps me a lot.* (P13)

*Then I felt safer, right? Having unprotected sex with my partner, even though I know he doesn’t only have sex with me. I feel safer.* (P13)

Being linked to a health service and being able to carry out regular outpatient clinical follow-up among PrEP users was mentioned as a benefit. The guidance provided by the healthcare team and the approach used also played an important role in adherence and maintenance of prophylaxis. Being in contact with the healthcare service and receiving information from the team was mentioned as a favorable factor for reflecting on habits and behaviors that influence health status.

*Now I think that precisely because I became a little freer, it allowed me to reevaluate my habits, my behaviors, and, let’s say, start a different life, so that today I even do it on demand, because I really don’t have the life I had before, right? From when I started.* (P3)

*Every three months, I have to come in and get tested, and see the doctor. Having this medical follow-up throughout my life is the biggest benefit for me.* (P14).

### Perceived barriers

The perceived barriers to PrEP use were related to individual, social, and structural aspects, as well as the need for regular medical appointments and difficulty reconciling them with work, difficulty scheduling appointments, linked to the complexity of the clinical protocol that requires follow-up visits and monitoring for laboratory tests, as well as difficulty in taking oral medication, side effects, and uncertainties about the long-term effects of the medication.

*I think what makes it difficult is the lack of openings at the BHU* [Basic Health Units]*. Doctors have schedules, they have everything, and sometimes we can’t get appointments. For example, I tried a few units nearby and there were no openings.* (P11)

*Taking pills every day. Trying to remember, for example, that men never had to take contraceptives like women do every day, so it’s more complicated for them to take, which is why I want to take the injection that’s coming out.* (P12)

*[...] I believe that now the doctor said there will be an injectable and it will be much better. This pill business is a pain.* (P15)

*[...] one disadvantage is that it is a new medication, no one knows what it will cause in 10 years.* (P9)

The stigma associated with PrEP use was also mentioned as a social barrier to PrEP use, which is still viewed as taboo and prejudiced by the community.

*There is this stigma attached to people who use PrEP, that they are more promiscuous, but anyway, I don’t care about this distorted opinion.* (P5)

*[...] the fact that they link PrEP with being promiscuous can be a disadvantage, but it would be more of a problem with society than with the medication itself.* (P14)

*I think it’s the judgment, right? Because it seems that those who take PrEP are promiscuous, sometimes they even are, but that’s when it would be interesting for them to take it. I think the LGBT community goes through a lot of things, and* [heterosexual] *men have a lot of advantages. [...] So I think that labeling someone else as promiscuous can end up inhibiting people from using PrEP, even though it’s a form of protection.* (P12)

## Discussion

The experiences of PrEP users are understood in light of HBM. These findings reveal that the search for PrEP was mainly motivated by perceived susceptibility to HIV acquisition, which constitutes a safer strategy among the various others adopted in people’s risk management.

The sociodemographic profile of participants is similar to that found in other countries such as Spain^(16)^, Australia^(17)^, and Belgium^(18)^. This fact reinforces that people seeking PrEP have a higher level of education and probably greater access to information that influences decision-making regarding their health situation^(19-20)^.

Although the search for this new preventive technology is encouraging, the literature points out that lower levels of education, stigmatization, and racism are associated with a lower intention to use and seek PrEP among black people and women^(21-22)^, which is a cause for concern, since in the Brazilian population, black people and those with low levels of education are the ones who register the highest number of AIDS cases^(23)^.

The HIV serology of partners was a relevant aspect of the use of PrEP. Being in a relationship with a partner living with HIV was identified as a motivation for using PrEP. This finding reinforces the participants’ perception of susceptibility to HIV acquisition and highlights PrEP as an effective strategy for preventing sexual transmission of the virus among serodiscordant couples. National^(24)^ and international^(25)^ guidelines prioritize these couples for primary HIV prevention, including biomedical strategies such as Treatment as Prevention (TasP) and PrEP.

Partnerships with different HIV serostatus have better results in HIV treatment and greater adherence to PrEP use^(26)^. This is because people living with HIV who are under medical supervision are encouraged to invite their partners to seek PrEP and to support each other in remembering to take their pills^(9)^. In addition, PrEP often acts as a bridge to ART among serodiscordant couples. From this perspective, the seronegative partner uses PrEP until the seropositive partner completes six months of regular ART use and reaches undetectable plasma viral load levels^(27)^.

Nevertheless, lack of knowledge or uncertainty about HIV serology among casual partners was also a reason for seeking prophylaxis in these findings. People living with HIV continue to consider disclosure of their serology a risky practice and live with their diagnosis in secret, for fear of the repercussions that this news could have on the community^(28-29)^.

Perceived susceptibility to HIV is also reflected in the possibility of failures or difficulties in condom use. Although condoms are an accessible, low-cost preventive method with no side effects, their effectiveness can be compromised by failures such as breakage or displacement. In addition, consistent condom use is a challenge for many people, with low adherence in the Brazilian population, including those most susceptible to HIV, such as young people, MSM, and sex workers^(30-31)^. A Brazilian study found that 85% of MSM reported inconsistent condom use and that the likelihood of inconsistent use increased among those who had steady partners, who practiced anal sex in the insertive position, or who had had an STI in the past^(32)^. These data highlight the need to raise awareness about the importance of condoms in preventing other STIs, especially among these populations.

In this context, PrEP was referred to as a viable method for those who do not use condoms consistently and was also considered a more effective strategy, providing greater safety and psychological well-being to its users. These findings are consistent with international studies, which show that reducing concern about the risk of transmission during sexual intercourse is directly related to improved sexual quality and satisfaction among couples^(33-35)^. Furthermore, as new prevention strategies emerge and become more popular, it is expected that the perception of HIV risk will not be based exclusively on condom use^(34)^.

People who perceive themselves to be at risk of HIV exposure due to uncertainty in relationships, even among stable partners, mentioned PrEP as a way to ensure self-care. This perception arises from a lack of certainty about the partner’s behavior, especially after experiences with other STIs or marital infidelity. The literature reports that couples in casual relationships face greater challenges in adopting prevention strategies due to a lack of intimacy to discuss these measures and the fact that many encounters occur unplanned^(36-37)^.

The findings of this study are noteworthy, indicating that individuals seek PrEP after being diagnosed with an STI, when they are in a relationship with an HIV-positive partner, or when they have multiple partners, in addition to reporting insecurity in using condoms alone as a prevention strategy. These results suggest that awareness campaigns about HIV risk factors and combination prevention are having a positive impact on the population. These individuals’ perception of risk reflects the factors that have been emphasized by health authorities and researchers in the field^(38)^.

The findings revealed the participants’ self-perception of risk in relation to various other potential STIs. This may be related to the fact that these STIs are not seen by people as health problems as serious as HIV. As a result, the information, motivation, and skills needed to mobilize the prevention of these infections may be treated differently^(39)^.

The social construction of HIV has evolved throughout history, incorporating different discourses and meanings. At the beginning of the epidemic, HIV was considered a “gay cancer”, seen as a death sentence, and continued to be surrounded by negative repercussions over time. Since then, several advances in the scientific field have allowed HIV to be managed as a chronic condition, resulting in longer survival and better quality of life for people living with the virus. However, HIV remains a virus with complex health repercussions, especially in cases of treatment failure^(40-41)^.

Some studies demonstrate the high effectiveness of PrEP as an HIV prevention strategy^(35,37,42)^ and, therefore, its continued use has had a positive impact on the experience of sexual activities. PrEP not only provides protection but also reduces the threat to relationships, alleviating fear and allowing the maintenance of emotional-sexual bonds^(43)^.

Furthermore, regardless of the HIV serology of sexual partners, PrEP is a viable alternative for those who do not adapt to regular condom use, as demonstrated in other studies^(34-35)^. Furthermore, the use of PrRP improves self-efficacy, making users feel more responsible and in control of sexual decisions, without depending on the honesty of their partners about their serological status^(35)^.

Another highlight was the clinical follow-up of PrEP, seen as an opportunity for health promotion, with frequent testing, continuous assessment, and regular requests for exams. Constant contact with the team was pointed out as a positive aspect, as it allows for reflection on habits and improvement of self-care among participants. These findings align with international studies, which indicated that PrEP users perceive the care package provide alongside preventive technology as a health advantage^(35,39)^.

The Ministry of Health^(24)^ recommends a set of interventions associated with the clinical monitoring of PrEP users, including general assessments, HIV testing, identification of other STIs such as chlamydia, syphilis, gonorrhea, and viral hepatitis, vaccination against Human Papillomavirus (HPV), as well as monitoring of renal function and pregnancy testing. Thus, the benefits of clinical monitoring of PrEP go beyond HIV prevention, as they enable early diagnosis of other infections, access to various preventive strategies, periodic health assessments, and serve as a gateway to the UHS.

In addition to health monitoring, building a positive interpersonal relationship with the team’s professionals was an important factor in adherence to prophylaxis. This reinforces that a welcoming environment, free of stigma and prejudice, fosters a stronger bond between users of preventive technology and the health service, thereby enhancing adherence to monitoring^(44)^.

Barriers to PrEP use involve individual, social, and structural aspects, as described in a literature review study^(9)^. Among the structural barriers, the complexity of the clinical protocol stands out, requiring regular follow-up and monitoring visits, including laboratory tests, in addition to long waiting times for appointments. These challenges were also identified in another study as barriers to the use of PrEP^(45)^.

Daily adherence to medication, especially among young people and men who are not in the habit of undergoing regular, long-term preventive monitoring, uncertainties about the side effects of long-term PrEP, and the need for frequent visits for clinical monitoring were identified as individual barriers to continued use of PrEP. Similar findings have been reported in other countries, where participants highlighted the difficulty of remembering to take the pills as a common challenge among PrEP users^(37)^.

After starting PrEP, the first dispensation will be for 30 days. Subsequent dispensations will occur every 90 days or, depending on medication adherence, every 120 days, as assessed by the healthcare professional. If the user does not receive the total amount of PrEP, they can obtain the remainder without needing a new HIV test, as long as it is within the validity period of the form. Users with continuous adherence for at least one year may have their clinical follow-up extended to six months, with adjustments to dispensing according to local availability. If they have symptoms of STIs, they should return to the health service^(24)^.

Oral antiretroviral regimens are highly effective for HIV prevention, with minimal toxicity and the convenience of a simple, once-daily dosing regimen. However, to address challenges related to adherence, long-acting alternatives such as injectable antiretrovirals, long-acting subcutaneous implants, and controlled-release vaginal rings have been studied. These options represent promising strategies, offering less frequent dosing, such as weekly oral agents or long-acting parenteral therapies. Such approaches may be particularly useful in situations where daily use of oral medications is difficult or treatment adherence is insufficient^(46)^.

Although many participants pointed to in-person follow-up as a challenge due to work schedules, new access strategies have emerged in the field of PrEP to overcome this barrier. These initiatives include online consultations, home delivery of pills, flexibility in medication pickup locations, and the inclusion of other professional categories as PrEP prescribers. These measures have facilitated access to PrEP and contributed to improving adherence and regularity in follow-up care^(24)^.

There are still opportunities to improve PrEP adherence through the implementation of personalized individual interventions and models that expand access, especially in non-traditional settings^(44)^. A review of the literature revealed that service delivery models are predominantly concentrated in clinics and hospitals focused on the treatment of STIs, and HIV, and clinics specializing in sexual health, especially in high-income countries^(47)^.

Nurses play a leading role in PrEP services in several countries and are key to expanding access to primary care, especially among vulnerable populations. Their work promotes health literacy about HIV PrEP, educating and monitoring people, managing services, and thus expanding access to and adherence to this prevention strategy. Nurse-led care models, such as the PrEP-RN clinic^(51-52)^, have shown that the relationship of trust and support provided to PrEP users contributes significantly to adherence. Thus, rethinking the service delivery model in Brazil, and strengthening the role of other trained health professionals, can reduce barriers to access and expand the reach of PrEP.

Self-management models have shown positive results, including the use of HIV self-tests and tests for other STIs, allowing people to replace in-person visits to clinics or laboratories with home self-care practices^(47)^. In addition, mobile health (mHealth) and digital health interventions have shown promise in HIV prevention, especially through smartphone apps. When integrated with PrEP services and combined with accessible testing, these technologies help overcome various barriers. They have the potential to anonymously reach and engage populations that are often left out of traditional prevention efforts while offering rapid, real-time delivery of programs at relatively low implementation costs^(49)^.

Knowledge about side effects and uncertainties regarding the long-term impacts of medication represent barriers for PrEP users. Although the benefits are widely recognized, the possible adverse effects of antiretrovirals are well documented, based on accumulated experience in their use in HIV treatment. However, a systematic review suggests that the risks of adverse events related to renal toxicity vary according to age and baseline creatinine clearance among PrEP users. In this context, less frequent or even optional screening for younger people without renal comorbidities may reduce barriers to the implementation and continued use of PrEP^(52)^.

This study can contribute to guiding person-centered care practices, promoting adherence to treatment by understanding their demands, and offering appropriate guidance and instructions. In addition, it reinforces the importance of developing campaigns, in partnership with the Ministry of Health, aimed at different audiences, intending to promote PrEP as an effective strategy for preventing and combating HIV.

The findings of this study highlight the central role of nursing in reducing health inequalities^(53)^, especially in access to PrEP. The analysis of the experiences of PrEP users highlights the relevance of risk perception and ongoing support to ensure adherence to preventive technology. The results highlight the need to invest in the daily practice of nursing professionals, focusing on vulnerable populations, reinforcing their ability to adapt sociocultural interventions, and leading initiatives aimed at health equity. It is recommended to strengthen the training and leadership of nurses, promoting the use of digital technologies and decentralized care models, aligned with social demands.

These findings should be interpreted in light of some limitations. First, the sample was homogeneous, composed predominantly of cisgender, white men who have sex with men. In addition, the data were collected in a large municipality in the interior of the state of São Paulo, which has distinct sociocultural and economic differences from other regions of Brazil.

## Conclusion

The study provided insight into the decisions, perceptions, benefits, and barriers to PrEP adherence and continuity from the perspective of people who use it. PrEP use was motivated by perceptions of susceptibility, serodiscordance, and lack of knowledge/uncertainty about the sexual partner’s HIV status. In addition, the severity of HIV infection and its association with incurable disease influenced health decisions. Among the benefits are protection against HIV, safety, and psychological well-being, as well as the opportunity for people to engage with a health service and undergo regular clinical and outpatient follow-up.

Barriers reflected individual, social, and structural aspects, such as difficulty in accessing and scheduling appointments, linked to the complexity of the clinical protocol, which requires follow-up visits and monitoring for laboratory tests, as well as difficulty in taking oral medication, side effects, and uncertainties about the long-term effects of the medication. Rethinking the model of PrEP service delivery for HIV prevention should include expanding the role of nurses in caring for users. It is essential that the approach be comprehensive, considering both individual and structural barriers, to strengthen the effectiveness of the strategy and advance the control of the HIV epidemic by 2030.

## Data Availability

Datasets related to this article will be available upon request to the corresponding author.
