# Supplementary material for: Motivations, benefits, and barriers to the use of HIV pre-exposure prophylaxis based on the health belief model
Source: Rev Lat Am Enfermagem. 2025 Nov 10;33:e4653. doi: 10.1590/1518-8345.7357.4653 (PMC12610931; doi:10.1590/1518-8345.7357.4653)
Supplement: Translation

# Translation: Motivações, benefícios e barreiras no uso da Profilaxia Pré-Exposição ao HIV fundamentados no modelo de crenças em saúde *

**DOI:** 10.1590/1518-8345.7357.4654

**Authors:** Rita de Cássia Pereira Rodrigues, Marcela Antonini, Laís do Espirito Santo Lima, Daniel de Macedo Rocha, Elucir Gir, Renata Karina Reis

**Affiliations:** 1 Universidade de São Paulo, Escola de Enfermagem de Ribeirão Preto, Centro Colaborador da OPAS/OMS para o Desenvolvimento da Pesquisa em Enfermagem, Ribeirão Preto, SP, Brasil Universidade de São Paulo Escola de Enfermagem de Ribeirão Preto Centro Colaborador da OPAS/OMS para o Desenvolvimento da Pesquisa em Enfermagem SP Ribeirão Preto Brasil

**Keywords:** HIV, Profilaxia Pré-Exposição, Prevenção de Doenças, Modelo de Crenças de Saúde, Enfermagem, Infecções Sexualmente Transmissíveis

## Abstract

compreender as motivações, benefícios e barreiras no uso da Profilaxia Pré-Exposição ao HIV.

estudo qualitativo fundamentado no Modelo de Crenças em Saúde. Participaram 16 pessoas usuárias da Profilaxia Pré-Exposição ao HIV acompanhadas em dois Centros de Testagem e Aconselhamento de um município de grande porte do interior do estado de São Paulo, Brasil. A coleta de dados foi realizada por entrevistas audiogravadas individuais. A análise de dados seguiu o Modelo de Crenças em Saúde de Rosenstock e a Análise de Conteúdo de Bardin.

a suscetibilidade ao HIV é influenciada pela incerteza sobre a sorologia dos parceiros e falhas no preservativo. A severidade do HIV é percebida como maior devido à sua incurabilidade. Os benefícios incluem maior proteção, segurança e bem-estar. As barreiras envolvem dificuldades de acesso, efeitos colaterais e estigma social.

o estudo revelou que a motivação para uso da Profilaxia Pré-Exposição foi pela percepção de suscetibilidade ao HIV, a gravidade da doença e benefícios como proteção e bem-estar psicológico, enquanto as barreiras envolveram dificuldades de acesso, complexidade do protocolo clínico e estigma. Conhecer as crenças e barreiras pode favorecer as intervenções mais efetivas pela equipe de saúde.

## Destaques

**(1)** Conhecer crenças de pessoas usuárias da Profilaxia Pré-Exposição favorece intervenções efetivas. **(2)** Reúne evidências favoráveis à prevenção combinada do HIV. **(3)** Reflete os desafios e barreiras enfrentadas por pessoas usuárias da Profilaxia Pré-Exposição. **(4)** Evidencia a necessidade de estratégias voltadas à informação em saúde.

## Introdução

Apesar dos progressos científicos, diagnósticos e terapêuticos, a infecção pelo Vírus da Imunodeficiência Humana (HIV) ainda representa um desafio contemporâneo e seu impacto epidemiológico, social, cultural, econômico e de saúde é amplamente documentado na literatura ^(1)^.Ao se considerar a magnitude do problema, o Conselho Geral das Nações Unidas propôs na Agenda para o Desenvolvimento Sustentável o controle da epidemia até o ano de 2030 a partir da informação e conscientização popular sobre as diferentes estratégias para a prevenção, como o uso da Profilaxia Pré-Exposição (PrEP) ^(2)^.

A PrEP é uma intervenção biomédica de prevenção do HIV altamente eficaz que representa um componente essencial da prevenção combinada recomendada pela Organização Mundial da Saúde (OMS) e implementada em diversos países desde 2015 ^(3)^.No Brasil, foi incorporada ao Sistema Único de Saúde (SUS) em 2017, sendo direcionada aos adultos e adolescentes sexualmente ativos sob risco aumentado para a infecção ^(4)^.Desde a sua implementação, 222.948 pessoas aderiram à estratégia. Apesar do crescimento no número de pessoas usuárias, a taxa de descontinuidade é elevada, refletindo desafios e barreiras para o uso e continuidade da PrEP ^(5)^.

Essa estratégia de prevenção combinada envolve o atendimento contínuo, que inclui a conscientização para o uso entre as pessoas com risco substancial de adquirir o HIV, a testagem regular, a captação, a adesão e a retenção no cuidado ^(6)^.Entretanto, as lacunas nessa modalidade assistencial são evidentes, sugerindo a necessidade de novas investigações para a compreensão dos determinantes, dos fatores associados e preditores para a adesão, assim como das barreiras e desafios na efetivação da estratégia e na carga desproporcional da infecção evidenciada entre os grupos em situação de vulnerabilidade ^(7-9)^.

Assim, a compreensão dos motivos para o uso da PrEP, dos benefícios e das potenciais barreiras enfrentadas para o uso desta estratégia entre as pessoas expostas ao HIV é fundamental para o desenvolvimento de intervenções eficazes para a melhoria do cuidado prestado a esta população. Dessa maneira, este estudo objetivou compreender as motivações, benefícios e barreiras no uso da PrEP ao HIV.

## Método

### Delineamento do estudo

Trata-se de um estudo descritivo de abordagem qualitativa conduzido conforme as diretrizes propostas pelo *Consolidated Criteria for Reporting Qualitative Research* (COREQ) ^(10)^ e fundamentado no Modelo de Crenças em Saúde (MCS) ^(11)^.

### Referencial teórico

O MCS foi desenvolvido em 1966 para explicar e compreender o comportamento humano no processo de saúde e no controle de doenças como o HIV ^(12-13)^.Trata-se de um modelo amplamente referenciado aplicado em pesquisa sobre os comportamentos de saúde e Enfermagem. Segundo o MCS, para uma pessoa agir preventivamente, ela precisa acreditar que: a) ela é suscetível a uma doença ou condição indesejável; b) sua ocorrência apresenta severidade para algum aspecto de sua vida; c) realizar uma intervenção profilática é eficaz para reduzir a suscetibilidade ou severidade da doença (benefícios); e d) a ação preventiva não implica em muitas barreiras ^(11)^.

As crenças de uma pessoa sobre esses fatores determinam sua probabilidade de se envolver em comportamento promotor da saúde e são utilizadas para explicar a adoção de comportamentos que possam auxiliar na prevenção e no controle das doenças, como o HIV, bem como identificar a aceitação de recomendações sobre os cuidados com a saúde ^(12-13)^.

A suscetibilidade percebida refere-se à percepção dos riscos subjetivos de contrair uma condição. A severidade percebida refere-se ao julgamento da pessoa em relação às implicações de um diagnóstico em sua vida, como por exemplo em seu trabalho, vida familiar ou relações sociais. Os benefícios são ações positivas e medidas preventivas relacionadas às consequências do diagnóstico. As barreiras estão relacionadas aos aspectos negativos, tais como: obstáculos, desconforto, gastos financeiros, entre outros ^(11)^.

### Local da coleta de dados

Os cenários do estudo foram dois Centros de Testagem e Aconselhamento (CTA) de um município de grande porte do estado de São Paulo, Brasil. Salienta-se que os CTA são unidades estratégicas no aconselhamento e diagnóstico precoce das Infecções Sexualmente Transmissíveis (ISTs/HIV) e o acesso a esses serviços para o atendimento está condicionado ao agendamento prévio e à consulta com uma equipe interdisciplinar para a testagem, orientação e aconselhamento.

Convém mencionar que o município estudado foi um dos pioneiros do país a implantar a PrEP na sua rede de atenção à saúde e, ao todo, possui cinco CTA, os quais estão distribuídos em cinco distritos sanitários. Assim, optou-se por selecionar os dois CTA que se constituíram nos primeiros locais de atendimento e dispensação da PrEP no município em questão, sendo um CTA (CTA-A) localizado em região de fácil acesso para a população que busca o atendimento para uso da PrEP, o qual possui pessoas em uso da PrEP em seguimento por mais tempo; e o outro CTA (CTA-B) está localizado em região do município que atende pessoas vivendo em áreas com maior vulnerabilidade socioeconômica. Buscou-se, portanto, incluir pessoas usuárias da PrEP com diferentes características sociodemográficas.

### Período

O estudo foi desenvolvido entre os meses de fevereiro e maio de 2023.

### População e critérios de seleção

No município estudado, a PrEP foi implementada em dezembro de 2017 e, até 2023, foram registradas 1.623 pessoas que usaram a PrEP, sendo que 1.199 tiveram pelo menos uma dispensação no SUS nos últimos 12 meses ^(4)^. Participaram do estudo pessoas soronegativas para o HIV, maiores de 18 anos, em uso da PrEP por no mínimo 90 dias e que estavam em acompanhamento no serviço de saúde. Casos novos de PrEP constituíram-se como critério de exclusão, pois considera-se que possíveis dificuldades de adesão são incipientes durante o início da profilaxia. A amostragem baseou-se no critério de conveniência intencional, ou seja, foram selecionados os participantes que estavam nos dias de coleta e que se enquadraram nos critérios estabelecidos para a inclusão.

### Participantes

Dezesseis participantes foram convidados a participar do estudo e não houve desistências ou recusas, sendo 13 atendidas no CTA-A e três no CTA-B. A seleção dos participantes buscou incluir homens e mulheres em uso de PrEP de diferentes faixas etárias, almejando contemplar pessoas com diferentes experiências, percepções e motivações.

### Coleta de dados

Os dados foram coletados por meio de entrevistas individuais e audiogravadas pela pesquisadora principal, que foi capacitada para este estudo. O roteiro foi composto por questões abertas e baseadas no MCS que abordavam os aspectos relacionados ao uso da PrEP. Realizou-se um estudo-piloto com quatro participantes visando analisar a viabilidade, adequação e revisão do questionário elaborado, bem como para fins de permitir a inserção da pesquisadora junto aos cenários e participantes do estudo. Destaca-se que os participantes da etapa-piloto não foram incluídos no estudo e o tempo médio estimado para as entrevistas foi de 15 minutos.

Após o piloto, o roteiro com perguntas relacionadas à experiência de usar a PrEP foi ajustado pelas pesquisadoras do estudo em conjunto com o grupo de pesquisa com *expertise* na temática, sendo adaptado para a sua versão final, que contemplou a caracterização dos participantes, assim como as seguintes questões norteadoras: Suscetibilidade percebida: O que te motivou a buscar a PrEP?; Severidade percebida: Por que você começou a usar a PrEP? Quais fatores influenciaram na adesão à PrEP?; Benefícios percebidos: Para você, quais são os benefícios de usar a PrEP?; Barreiras percebidas: Para você, quais as barreiras/desvantagens de usar a PrEP?. Para resguardar a confidencialidade dos dados, os participantes foram identificados com a letra “P” designando o “Participante” juntamente com sua ordem de inclusão (P1, P2 até P16).

A coleta de dados foi realizada com a devida autorização da Secretaria de Saúde do município. A pesquisa foi aprovada pelo Comitê de Ética em Pesquisa (CEP), bem como acordada com os responsáveis pelos CTA. Foi disponibilizada uma sala privativa e silenciosa para a realização das entrevistas, que ocorreram semanalmente, no horário das 8h às 17h, as quais foram conduzidas pela pesquisadora principal nos dias de agendamento das pessoas usuárias da PrEP para o atendimento pelas equipes de saúde.

Os participantes que estavam nos CTA para a consulta de retorno referente ao uso da PrEP que, possivelmente, se enquadraram nos critérios de inclusão definidos no estudo, foram abordados individualmente pela pesquisadora principal, a qual se apresentava e explicava os objetivos do estudo em sala privativa, antes ou após a consulta médica. Após a concordância por escrito dos participantes, mediante assinatura do Termo de Consentimento Livre e Esclarecido (TCLE), a pesquisadora realizava as entrevistas individuais, com auxílio do roteiro e do gravador de voz.

### Análise e tratamento dos dados

Todas as entrevistas foram transcritas na íntegra e a coleta de dados foi realizada até a constatação da saturação dos dados discutida entre os autores ^(14)^.A análise dos dados foi norteada pelo MCS de Rosenstock ^(11)^. Realizou-se a Análise de Conteúdo, conforme proposto por Bardin ^(15)^, considerando as seguintes etapas para organização dos dados: pré-análise, exploração do material e interpretação, com tratamento por codificação. A codificação e validação foi realizada pelos próprios autores, sem o uso de *softwares*, respeitando a “árvore de códigos” de temas agrupados em significados temáticos para a posterior geração de categorias. Destaca-se que as categorias foram previamente definidas baseadas no modelo teórico adotado no estudo.

### Aspectos éticos

O projeto foi aprovado pela Secretaria de Saúde do município estudado e pelo CEP da instituição responsável, sob o parecer nº 4.852.588, de 16/07/2021. Todos os participantes foram informados sobre os objetivos, benefícios e riscos do estudo, com posterior assinatura do TCLE.

## Resultados

A amostra foi composta por 16 participantes, com idade média de 36,5 anos. A maioria era do sexo masculino, representando 15 (93,75%) participantes, dos quais 13 (81,25%) eram Homens que faziam Sexo com Homens (HSH) e 10 (62,50%) eram solteiros. Quanto ao tipo de parceria, cinco (31,25%) tinham uma parceria fixa, enquanto 10 (62,50%) mantinham parcerias casuais. Em relação à escolaridade, nove (56,25%) tinham Ensino Médio completo, três (18,75%) possuíam graduação, um (6,25%) tinha pós-graduação e outro (6,25%) possuía curso técnico.

As categorias resultantes do processo de análise respeitaram as dimensões do MCS, sendo expressas por 22 temas. Ao refletir sobre o uso da PrEP para o HIV, os participantes reconheceram que, embora exista tratamento, não há cura. Ainda, reconheceram os benefícios advindos da medicação e do acompanhamento clínico. No entanto, dificuldades estão presentes, relacionando-se à acessibilidade ao serviço de saúde, estigma, efeitos colaterais e incertezas sobre as repercussões da medicação. As dimensões do modelo proposto, assim como os temas que compõem cada domínio, estão descritos na Figura 1.

**Figura 1-:** Temas emergentes de acordo com cada dimensão do Modelo de Crenças em Saúde. Ribeirão Preto, SP, Brasil, 2023

*HIV = Vírus da Imunodeficiência Humana; ^†^ ISTs = Infecções Sexualmente Transmissíveis; ^‡^ PrEP = Profilaxia Pré-exposição

### Suscetibilidade percebida

A percepção adequada das pessoas quanto à suscetibilidade para a aquisição do HIV motivou a decisão da busca pela PrEP. Diversos fatores foram identificados, como estar em um relacionamento com uma pessoa que vive com o HIV, ou o desconhecimento/incerteza da sorologia anti-HIV da parceria sexual.

*Porque no caso ela* [a parceira] *contém a aids, ela tem o HIV, ela não tem a doença, só o vírus, e “aí” é um modo preventivo, como ela tem, ela faz tratamento e eu tenho que fazer junto com ela.* (P1)

*A princípio de tudo, eu conheci por conta do relacionamento anterior que eu tinha, por conta do HIV dele, aí quando eu fiquei solteiro eu continuei tomando porque achei mais viável de me proteger, além do preservativo, e agora com o novo relacionamento sorodiscordante, o que me motiva é a questão da prevenção mesmo, acho melhor ter esse cuidado adicional.* (P15)

*Não foi só por conta desse relacionamento, mas para outros, ninguém vem com uma plaquinha escrito que tem HIV na testa e nem todo mundo tem a liberdade de falar.* (P6)

Para os participantes, o uso da PrEP confere maior segurança e liberdade contra a exposição e potencial aquisição do HIV, de modo que o seu uso foi motivado também pela percepção sobre os limites do preservativo como método de prevenção em função de situações envolvendo acidentes como o rompimento, ou até mesmo pela resistência e dificuldade de negociação do preservativo ou não gostar de utilizá-lo com a parceria.

[...] *e nem todo mundo tem a liberdade de falar, aí se caso acontecesse algum acidente, eu uso os outros métodos, mas estaria mais exposto.* (P12)

*Bom, na verdade assim, a gente fica um pouquinho sem jeito para falar disso, mas mesmo na relação com camisinha sempre ficava, como que eu vou te dizer, uma desconfiança, um medo, uma sensação de que eu também poderia ter contraído HIV, por exemplo. E, também tem a outra questão de não gostar da relação com preservativo, entendeu?* (P8)

*Pra me sentir mais segura, pra me sentir protegida, porque ele* [o parceiro] não consegue ter relação com preservativo, e eu estava me sentindo muito exposta. (P13)

A suscetibilidade para a aquisição do HIV também foi descrita na incerteza dos relacionamentos mesmo entre as parcerias fixas, por não ter garantia do comportamento do outro e, também nas situações concretas após vivenciar a aquisição de outras IST ou traição conjugal.

*Porque eu acho que é uma proteção a mais, caso a camisinha tenha rompido, e eu até pensei se caso eu ter um parceiro fixo, a gente nunca sabe se vai ser traído ou não, então pelo menos do HIV eu sei que estaria* okay *.* (P12)

*O que me motivou foi que eu já tive uma IST, e a primeira vez que eu fui fazer exame eu fiquei em pânico. Aí eu pensei assim que eu teria que me cuidar, que eu preciso ter um acompanhamento e me cuidar mais.* (P14)

*Meu marido me traiu, então eu percebi que não tem como confiar em qualquer pessoa.* (P4)

A suscetibilidade percebida de aquisição do HIV também foi identificada entre aqueles que referiam múltiplas parcerias sexuais ou eram profissionais do sexo.

*Porque como eu não tenho parceiro fixo, a exposição é muito grande, e sendo assim, as chances de contrair o vírus é muito grande também, então, por isso eu dei entrada pra tá me protegendo do vírus por meio da PrEP.* (P7)

*Quando a gente entra na prostituição, a gente entra pra ter lucros, ganhar o seu dinheiro, se manter. Pra mim é uma profissão bem legal, normal, porque através da prostituição eu conhecia muita gente boa, consegui minhas coisas. Eu tinha muito medo de pegar a doença, e, também na prostituição existem preços de serviços,* né *? E tem serviços que são bem mais lucrativos que os outros, e eu não fazia, então acabava sempre ficando para trás, muito limitada. E aí depois da PrEP eu consegui.* (P15)

### Severidade percebida

Para os entrevistados, a infecção pelo HIV pode lhes trazer danos à saúde, já que a doença tem tratamento mas é incurável. Diferentemente, observa-se uma percepção de menor risco ou severidade em caso de aquisição de outras IST.

*Então, eu fui mais por conta dela, porque eu não sabia que existia prevenção, eu sabia que existia tratamento “né?”, do HIV, e como ela tinha que tratar, eu procurei onde, “aí” eu vim tratar e como nós éramos parceiros, falaram que eu tinha que fazer o tratamento junto, porque não tem cura ainda, então vai ir até durar nossas vidas.* (P1)

*Há outras* [referindo-se a outras IST], *com certeza, tudo volta à estaca zero,* né *? Sei que eu estou protegido se você pensa na hepatite, que tem as vacinas e ‘tal’. Mas é muito maior* [referindo-se ao HIV] *e eu tenho muito mais medo.* (P1)

### Benefícios percebidos

Os benefícios identificados com o uso da PrEP foram o sentimento de maior proteção contra o HIV e autonomia, trazendo como consequência os sentimentos de melhor segurança e bem-estar psicológico. Sentir-se seguro permitiu que as pessoas desfrutassem de suas vidas sexuais de forma mais prazerosa.

*Acho uma libertação, uma segurança a mais, uma oportunidade a mais na vida.* (P5)

*A segurança mesmo,* né *? Da prevenção combinada, de ter mais um meio de prevenção contra o HIV.* (P16)

[...] *o principal benefício é essa questão de não vir aquela preocupação da possibilidade de se contaminar* [...]. (P3)

*Ter um pouco mais de segurança,* né *? Porque acidentes acontecem, então usar a PrEP me deixa um pouco mais confiante em relação ao risco de me contaminar.* (P12)

*O benefício também é que eu me sinto com a minha mente mais tranquila, não fico com aquele pesar de chegar aqui e ter um positivo me esperando. Então em relação a isso é o que ela me ajuda bastante.* (P13)

*Aí eu fiquei mais seguro,* né *? De ter uma relação desprotegida com o meu parceiro, mesmo sabendo que ele não tem relações só comigo. Me sinto mais seguro.* (P13)

Estar vinculado a um serviço de saúde e poder realizar o acompanhamento clínico ambulatorial regular entre as pessoas usuárias da PrEP foi referido como um benefício. A orientação da equipe de saúde e a abordagem utilizada também teve papel importante na adesão e na manutenção da profilaxia. Estar em contato com o serviço de saúde e receber as informações da equipe foi referido como um movimento propício para refletir sobre os hábitos e comportamentos que influenciam na situação de saúde.

*Agora eu acho que justamente por conta de eu ter ficado um pouco mais livre, isso me permitiu reavaliar os meus hábitos, meus comportamentos, e digamos assim, começar uma vida diferente, de forma que hoje eu até mesmo faço ela sob demanda, porque realmente eu não tenho mais a vida que eu tinha antes,* né *? De quando eu comecei.* (P3)

*Eu tenho de três em três meses vir e ser testado, passar com o médico. Ter esse acompanhamento médico ao longo da vida é o maior benefício pra mim.* (P14).

### Barreiras percebidas

As barreiras percebidas para o uso da PrEP foram tanto relacionadas aos aspectos individuais, sociais e estruturais, quanto à necessidade de consultas médicas regulares e dificuldade de conciliar com o trabalho, dificuldade de agendamento das consultas, atreladas com a complexidade do protocolo clínico que prescinde da necessidade das visitas de acompanhamento e monitorização para a realização de exames laboratoriais, além de dificuldade de ingestão dos medicamentos por via oral, efeitos colaterais e incertezas sobre os efeitos da medicação a longo prazo.

*Eu acho que o que dificulta é a falta de vagas nas UBS* [Unidades Básicas de Saúde]. *Que os médicos t* ê *m agenda, tem tudo, e às vezes a gente não consegue as vagas. Por exemplo, aqui perto eu tentei algumas unidades e aí não tinha vaga.* (P11)

*Usar comprimido todo dia. De tentar lembrar, por exemplo, o homem nunca precisou tomar anticoncepcional que nem a mulher todos os dias, então para ele é mais complicado de tomar, por isso que eu quero tomar a injeção que vai sair.* (P12)

[...] *acredito que agora a doutora disse que vai ter um injetável e vai ser bem melhor. Que esse negócio de comprimido é um porre.* (P15)

[...] *uma desvantagem é que é uma medicação nova, ninguém sabe o que ela vai ocasionar daqui a 10 anos.* (P9)

O estigma associado ao uso da PrEP também foi mencionado como uma barreira social, já que seu uso ainda é visto como tabu e com preconceito pela comunidade.

*Existe esse estigma de quem usa PrEP, de quem é mais promíscuo, mas enfim, não me importo com essa opinião distorcida.* (P5)

*[...] o fato de ligarem a PrEP com ser promíscuo pode ser uma desvantagem, mas seria mais um problema da sociedade do que do medicamento em si.* (P14)

*Eu acho que o julgamento,* né *? Porque parece que quem toma PrEP é promíscua, às vezes até é mesmo, mas aí que seria interessante ela tomar. Eu acho que a comunidade LGBT passa por uma série de coisas, e o homem [heterossexual] tem muita vantagem. [...] então eu acho que alguém tachar outra pessoa de promíscuo pode acabar inibindo as pessoas de usarem a PrEP, mesmo sendo uma proteção.* (P12)

## Discussão

Compreende-se as experiências de pessoas usuárias de PrEP à luz do MCS. Estes achados revelam que a busca pela PrEP foi motivada principalmente pela suscetibilidade percebida de aquisição do HIV, e que configura uma estratégia de maior segurança dentre as diversas outras que são adotadas na gestão de risco das pessoas.

O perfil sociodemográfico dos participantes assemelha-se ao encontrado em outros países como Espanha ^(16)^, Austrália ^(17)^ e Bélgica ^(18)^. Tal fato reforça que as pessoas que procuram a PrEP detêm um maior nível de escolaridade e provavelmente maior acesso às informações que influenciam na tomada de decisões para sua situação de saúde ^(19-20)^.

Apesar de animadora a busca dessa nova tecnologia preventiva, a literatura aponta que menor nível de escolaridade, estigmatização e racismo estão associados à menor intenção de uso e busca pela PrEP por pessoas negras e mulheres ^(21-22)^, configurando uma preocupação, uma vez que, na população brasileira, pessoas negras e de baixa escolaridade são as que registram maior número de casos de aids ^(23)^.

A sorologia anti-HIV das parcerias foi um aspecto relevante para o uso da PrEP. Estar em um relacionamento com alguma parceria que vive com o HIV foi identificado como motivação para o uso da PrEP. Esse achado reforça a percepção dos participantes quanto à suscetibilidade percebida à aquisição do HIV, além de destacar a PrEP como uma estratégia eficaz para a prevenção da transmissão sexual do vírus entre os casais sorodiferentes. As diretrizes nacionais ^(24)^ e internacionais ^(25)^ priorizam esses casais para a prevenção primária do HIV, incluindo estratégias biomédicas como o Tratamento como Prevenção (TasP) e a PrEP.

As parcerias com sorologias anti-HIV diferentes apresentam melhores resultados no tratamento do HIV e maior adesão ao uso da PrEP ^(26)^.Isso se deve ao fato de que as pessoas vivendo com HIV, que estão em acompanhamento médico, são incentivadas a convidar seus parceiros a buscar a PrEP e a se apoiarem mutuamente para lembrar de tomar as pílulas ^(9)^. Além disso, a PrEP frequentemente funciona como uma ponte para a terapia antirretroviral (TARV) entre os casais sorodiferentes. Sob essa perspectiva, a parceria soronegativa faz uso da PrEP até que a parceria soropositiva complete seis meses em uso regular de TARV e atinja os níveis indetectáveis da carga viral plasmática ^(27)^.

Não obstante, o desconhecimento ou incerteza da sorologia anti-HIV entre as parcerias casuais também configurou-se motivo para a busca da profilaxia nestes achados. Pessoas que vivem com HIV continuam considerando a revelação de sua sorologia uma prática arriscada e convivem com o diagnóstico em segredo, por medo da repercussão que esta notícia poderia ter na comunidade ^(28-29)^.

A suscetibilidade percebida ao HIV também se reflete na possibilidade de falhas ou dificuldades no uso do preservativo. Embora o preservativo seja um método preventivo acessível, de baixo custo e sem efeitos colaterais, sua efetividade pode ser comprometida por falhas como rompimento ou deslocamento. Além disso, o uso consistente do preservativo é um desafio para muitas pessoas, com baixa adesão na população brasileira, incluindo aquelas mais suscetíveis ao HIV como jovens, HSH e profissionais do sexo ^(30-31)^. Um estudo brasileiro identificou que 85% dos HSH relataram uso inconsistente do preservativo, e que a probabilidade de uso inconsistente aumentava entre aqueles que tinham parcerias fixas, que praticavam sexo anal em posição insertiva ou que já haviam tido uma IST no passado ^(32)^.Esses dados ressaltam a necessidade de intensificar a conscientização sobre a importância do preservativo na prevenção de outras ISTs, especialmente entre essas populações.

Nesse contexto, a PrEP foi referida como um método viável para aqueles que não utilizam o preservativo de forma consistente, sendo também considerada uma estratégia mais eficaz, que proporciona maior segurança e bem-estar psicológico aos seus usuários. Esses achados são consistentes com estudos internacionais, que demonstram que a redução da preocupação com o risco de transmissão durante o ato sexual está diretamente relacionada à melhoria da qualidade e satisfação sexual entre os casais ^(33-35)^.Além disso, à medida que novas estratégias de prevenção emergem e se tornam mais populares, é esperado que a percepção do risco de aquisição do HIV não seja pautada exclusivamente ao uso do preservativo ^(34)^.

Pessoas que se percebem em risco de exposição ao HIV devido à incerteza nos relacionamentos, mesmo entre parcerias fixas, mencionaram a PrEP como uma forma de garantir o autocuidado. Essa percepção surge da falta de garantia sobre o comportamento do parceiro, especialmente após as experiências com aquisição de outras ISTs ou infidelidade conjugal. A literatura relata que os casais em relacionamentos casuais enfrentam maiores desafios na adoção de estratégias de prevenção, devido à falta de intimidade para discutir essas medidas e ao fato de muitos encontros ocorrerem de maneira não planejada ^(36-37)^.

São dignos de atenção os achados deste estudo, que indicam a busca pela PrEP após o diagnóstico de uma IST, ou se relacionar com uma parceria de sorologia positiva para o HIV, ou por ter múltiplas parcerias, além de relatar a insegurança em utilizar apenas o preservativo como estratégia de prevenção. Esses resultados sugerem que as campanhas de conscientização sobre os fatores de risco para o HIV e sobre a prevenção combinada estão alcançando um impacto positivo na população. A percepção de risco dessas pessoas reflete os fatores que, de fato, têm sido enfatizados por autoridades em saúde e pesquisadores da área ^(38)^.

Os achados revelaram a autopercepção de risco dos participantes em relação às diversas outras potenciais ISTs. Isso pode estar relacionado ao fato de que essas ISTs não são vistas pelas pessoas como problemas de saúde tão graves quanto o HIV. Como resultado, a informação, a motivação e as competências necessárias para mobilizar a prevenção dessas infecções podem ser tratadas de forma diferente ^(39)^.

A construção social do HIV evoluiu ao longo da história, incorporando diferentes discursos e significados. No início da epidemia, o HIV foi considerado um “câncer gay”, visto como uma sentença de morte, e continuou sendo cercado de repercussões negativas ao longo do tempo ^(40)^.Desde então, diversos avanços no campo científico permitiram que o HIV fosse manejado como uma condição crônica, resultando em maior sobrevida e qualidade de vida para as pessoas que vivem com o vírus. No entanto, o HIV continua sendo um vírus com repercussões complexas para a saúde, especialmente em casos de falha no tratamento ^(40-41)^. 

Alguns estudos demonstram a alta efetividade da PrEP como estratégia de prevenção ao HIV ^(35, 37, 42)^ e, por isso, o seu uso contínuo tem impactado positivamente na experiência das atividades sexuais. A PrEP não apenas proporciona proteção, mas também reduz a ameaça ao relacionamento, aliviando o medo e permitindo a manutenção dos vínculos afetivo-sexuais ^(43)^.

Além disso, independentemente da sorologia anti-HIV das parcerias sexuais, a PrEP se apresenta como uma alternativa viável para aqueles que não se adaptam ao uso regular do preservativo, como demonstrado em outros estudos ^(34-35)^.Adicionalmente, o uso da PrRP melhora a autoeficácia, fazendo com que as pessoas usuárias se sintam mais responsáveis e no controle das decisões sexuais, sem depender da honestidade de suas parcerias sobre seu estado de sorológico ^(35)^.

Outro ponto destacado foi o seguimento clínico da PrEP, visto como uma oportunidade de promoção da saúde, com testagens frequentes, avaliação contínua e solicitação regular de exames. O contato constante com a equipe foi apontado como um aspecto positivo, pois possibilita a reflexão sobre os hábitos e o aprimoramento do autocuidado entre os participantes. Esses achados estão alinhados com estudos internacionais, que mostram que as pessoas usuárias de PrEP reconhecem o pacote de cuidados oferecidos juntamente com a tecnologia preventiva como uma vantagem para a saúde ^(35, 39)^.

O Ministério da Saúde ^(24)^ recomenda um conjunto de intervenções associadas ao acompanhamento clínico de pessoas usuárias de PrEP, incluindo avaliações gerais, testagem para HIV, identificação de outras ISTs, como clamídia, sífilis, gonorreia e hepatites virais, vacinação contra o Papiloma Vírus Humano (HPV), além de monitoramento da função renal e teste de gravidez. Assim os benefícios do acompanhamento clínico da PrEP vão além da prevenção do HIV, pois possibilitam o diagnóstico precoce de outras infecções, acesso às diversas estratégias preventivas, avaliação periódica da saúde, além de ser uma porta de entrada para o SUS.

Além do acompanhamento em saúde, a construção de uma relação interpessoal positiva com os profissionais da equipe foi um fator importante para a adesão à profilaxia. Isso reforça que um ambiente acolhedor e um atendimento livre de estigmas e preconceitos favorecem o vínculo das pessoas usuárias da tecnologia preventiva com o serviço de saúde, fortalecendo, assim, a adesão ao acompanhamento ^(44)^.

As barreiras para o uso da PrEP envolvem aspectos individuais, sociais e estruturais, conforme descrito em estudo de revisão da literatura ^(9)^. Entre as barreiras estruturais, destacam-se a complexidade do protocolo clínico, que exige visitas regulares de acompanhamento e monitorização, incluindo a realização de exames laboratoriais, além do tempo prolongado de espera para as consultas. Esses desafios também foram identificados em outro estudo como barreiras ao uso da PrEP ^(45)^.

A adesão diária aos medicamentos, especialmente entre os jovens e os homens que não possuem o hábito de realizar acompanhamento preventivo regular e de longo prazo, as incertezas dos efeitos colaterais da PrEP de longo prazo e a necessidade de visitas frequentes para o acompanhamento clínico foram identificadas como barreiras individuais à continuidade do uso da PreP. Achados semelhantes foram relatados em outros países, onde os participantes destacaram a dificuldade em lembrar de tomar as pílulas como um desafio comum entre as pessoas usuárias de PrEP ^(37)^.

Após iniciar a PrEP, a primeira dispensação será de 30 dias. As dispensações seguintes ocorrerão a cada 90 dias ou, dependendo da adesão à medicação, a cada 120 dias, conforme a avaliação do profissional de saúde. Se a pessoa usuária não receber a quantidade total de PrEP, ela pode obter o restante sem precisar de novo teste de HIV, desde que dentro da validade do formulário. Pessoas usuárias com adesão contínua por pelo menos um ano podem ter o acompanhamento clínico ampliado para seis meses, com ajuste na dispensação conforme a disponibilidade local. Caso apresentem sintomas de IST, devem retornar ao serviço de saúde ^(24)^.

Os regimes antirretrovirais orais são altamente eficazes para a prevenção do HIV, apresentando toxicidade mínima e a conveniência de uma dosagem simples, com um único comprimido diário. No entanto, para abordar os desafios relacionados à adesão, têm sido estudadas alternativas de ação prolongada, como antirretrovirais injetáveis, implantes subcutâneos de longa duração e anéis vaginais de liberação controlada. Essas opções representam estratégias promissoras, oferecendo dosagens menos frequentes, como agentes orais administrados semanalmente ou terapias de ação prolongada por via parenteral. Tais abordagens podem ser particularmente úteis em situações em que o uso diário de medicamentos orais é difícil ou a adesão ao tratamento é insuficiente ^(46)^.

Embora muitos participantes tenham apontado o acompanhamento presencial como um desafio devido aos horários de trabalho, novas estratégias de acesso têm surgido no campo da PrEP para superar essa barreira. Entre essas iniciativas estão as consultas *online*, entrega domiciliar das pílulas, flexibilização dos locais de retirada da medicação e a inclusão de outras categorias profissionais como prescritores de PrEP. Essas medidas têm facilitado o acesso à PrEP e contribuído para melhorar a adesão e a regularidade nos cuidados de acompanhamento ^(24)^.

Há ainda oportunidades para melhorar a adesão à PrEP por meio da implementação de intervenções individuais personalizadas e de modelos que ampliem o acesso, especialmente em ambientes não tradicionais ^(44)^. Uma revisão da literatura revelou que os modelos de prestação de serviços estão predominantemente concentrados em clínicas e hospitais voltados para o tratamento de ISTs, HIV e clínicas especializadas em saúde sexual, especialmente em países de alta renda ^(47)^.

Os enfermeiros desempenham um papel de liderança em serviços de PrEP ^(48)^ em diversos países, sendo fundamentais para ampliar o acesso aos cuidados primários, especialmente entre as populações vulnerabilizadas ^(49)^. Sua atuação promove a alfabetização em saúde sobre a PrEP do HIV, educando e monitorando pessoas, gerindo serviços, ampliando, portanto, o acesso e a adesão a essa estratégia de prevenção ^(50)^. Modelos de cuidado liderados por enfermeiros, como a clínica PrEP-RN ^(51-52)^, demonstraram que a relação de confiança e o suporte proporcionados às pessoas usuárias de PrEP contribuem significativamente para a adesão. Assim, repensar o modelo de prestação de serviços no Brasil, fortalecendo o papel de outros profissionais de saúde capacitados, pode reduzir barreiras de acesso e ampliar o alcance da PrEP.

Modelos voltados à autogestão têm demonstrado resultados positivos, incluindo o uso de autotestes para o HIV e testes para outras ISTs, permitindo que as pessoas substituam as visitas presenciais a clínicas ou laboratórios por práticas de autocuidado domiciliar ^(47)^.Além disso, intervenções de saúde móvel ( *mHealth* ) e saúde digital têm se mostrado promissoras na prevenção do HIV, especialmente por meio de aplicativos para *smartphones.* Essas tecnologias, quando integradas aos serviços de PrEP e associadas aos testes acessíveis, ajudam a superar as diversas barreiras. Elas têm o potencial de alcançar e envolver anonimamente as populações que muitas vezes ficam fora dos esforços tradicionais de prevenção, além de oferecerem entrega rápida e em tempo real de programas, com custos de implementação relativamente baixos ^(49)^.

O conhecimento sobre os efeitos colaterais e as incertezas quanto aos impactos da medicação a longo prazo representam barreiras para as pessoas usuárias de PrEP. Embora os benefícios sejam amplamente reconhecidos, os possíveis efeitos adversos dos antirretrovirais são bem documentados, com base na experiência acumulada em seu uso no tratamento do HIV. No entanto, uma revisão sistemática sugere que os riscos de eventos adversos relacionados à toxicidade renal variam conforme a idade e a depuração basal de creatinina entre as pessoas usuárias de PrEP. Nesse contexto, a realização de rastreamento menos frequente ou mesmo opcional para pessoas mais jovens sem comorbidades renais pode reduzir barreiras à implementação e ao uso contínuo da PrEP ^(52)^.

Este estudo pode contribuir para orientar as práticas de cuidado centradas na pessoa, favorecendo a adesão ao tratamento ao compreender suas demandas e oferecer as orientações e instruções adequadas. Além disso, reforça a importância de desenvolver campanhas, em parceria com o Ministério da Saúde, voltadas aos diferentes públicos, com o objetivo de promover a PrEP como estratégia eficaz de prevenção e combate ao HIV.

Os achados deste estudo ressaltam o papel central da Enfermagem na redução das desigualdades em saúde ^(53)^, especialmente no acesso à PrEP. A análise das experiências das pessoas usuárias de PrEP evidencia a relevância da percepção de risco e do suporte contínuo para garantir a adesão à tecnologia preventiva. Os resultados destacam a necessidade de investir na prática cotidiana dos profissionais de enfermagem, com foco em populações vulnerabilizadas, reforçando sua habilidade de adaptar intervenções socioculturais e liderar as iniciativas voltadas à equidade em saúde. Recomenda-se fortalecer a formação e a liderança dos enfermeiros, promovendo o uso de tecnologias digitais e de modelos de cuidados descentralizados, alinhados às demandas sociais.

Estes achados devem ser interpretados à luz de algumas limitações. Primeiramente, a amostra foi homogênea, composta predominantemente por homens cisgênero, brancos e que fazem sexo com homens. Adicionalmente, os dados foram coletados em um município de grande porte no interior do estado de São Paulo, que apresenta características socioculturais e econômicas distintas de outras regiões do Brasil.

## Conclusão

O estudo possibilitou compreender as decisões, percepções, benefícios e barreiras para a adesão e a continuidade da PrEP, na perspectiva das pessoas que a usam. O uso da PrEP foi motivado pela percepção da suscetibilidade, da sorodiferença e do desconhecimento/incerteza da sorologia anti-HIV da parceria sexual. Além disso, a gravidade da infecção pelo HIV e sua associação como doença incurável influenciaram as decisões em saúde. Dentre os benefícios estão a proteção contra o HIV, a segurança e o bem-estar psicológico, assim como a possibilidade de a pessoa se vincular a um serviço de saúde e realizar o acompanhamento clínico-ambulatorial de forma regular.

Quanto às barreiras, estas refletiram aspectos individuais, sociais e estruturais como a dificuldade de acesso e agendamento das consultas, atreladas à complexidade do protocolo clínico que prescinde da necessidade das visitas de acompanhamento e monitorização para a realização de exames laboratoriais, além da dificuldade de ingestão dos medicamentos por via oral, dos efeitos colaterais e das incertezas sobre os efeitos da medicação a longo prazo. Repensar o modelo de prestação de serviços de PrEP para a prevenção do HIV deve incluir a ampliação do papel do enfermeiro no atendimento às pessoas usuárias. É essencial que a abordagem seja integral, considerando tanto as barreiras individuais quanto as estruturais, de modo a fortalecer a efetividade da estratégia e avançar no controle da epidemia de HIV até 2030.

## Data Availability

Os conjuntos de dados relacionados a este artigo estarão disponíveis mediante solicitação ao autor correspondente.

# Translation: Motivaciones, beneficios y barreras en el uso de la Profilaxis Preexposición al VIH fundamentados en el modelo de creencias en salud*

**DOI:** 10.1590/1518-8345.7357.4652

**Authors:** Rita de Cássia Pereira Rodrigues, Marcela Antonini, Laís do Espirito Santo Lima, Daniel de Macedo Rocha, Elucir Gir, Renata Karina Reis

**Affiliations:** 1 Universidade de São Paulo, Escola de Enfermagem de Ribeirão Preto, Centro Colaborador de la OPS/OMS para el Desarrollo de la Investigación en Enfermería, Ribeirão Preto, SP, Brasil Universidade de São Paulo Escola de Enfermagem de Ribeirão Preto Centro Colaborador de la OPS/OMS para el Desarrollo de la Investigación en Enfermería SP Ribeirão Preto Brasil

**Keywords:** VIH, Profilaxis Pre-Exposición, Prevención de Enfermedades, Modelo de Creencias sobre la Salud, Enfermería, Enfermedades de Transmisión Sexual

## Abstract

comprender las motivaciones, beneficios y barreras en el uso de la Profilaxis Preexposición al VIH.

estudio cualitativo fundamentado en el Modelo de Creencias en Salud. Participaron 16 personas usuarias de la Profilaxis Preexposición al VIH atendidas en dos Centros de Pruebas y Asesoramiento de un municipio de gran tamaño del interior del estado de São Paulo, Brasil. La recolección de datos se realizó mediante entrevistas individuales grabadas en audio. El análisis de datos siguió el Modelo de Creencias en Salud de Rosenstock y el Análisis de Contenido de Bardin.

la susceptibilidad al VIH está influenciada por la incertidumbre sobre la serología de las parejas y fallos en el uso del preservativo. La severidad del VIH es percibida como alta debido a su carácter incurable. Los beneficios incluyen mayor protección, seguridad y bienestar. Las barreras envuelven dificultades de acceso, efectos secundarios y estigma social.

el estudio reveló que la motivación para el uso de la Profilaxis Preexposición se relaciona con la percepción de susceptibilidad al VIH, la gravedad de la enfermedad y beneficios como protección y bienestar psicológico, mientras que las barreras implican dificultades de acceso, complejidad del protocolo clínico y estigmatización. Conocer las creencias y barreras puede favorecer intervenciones más efectivas por parte del equipo de salud.

## Destacados

**(1)** Conocer las creencias de las personas usuarias de la Profilaxis Preexposición puede favorecer la implementación de intervenciones efectivas. **(2)** Reúne evidencias favorables a la prevención combinada del VIH. **(3)** Refleja los desafíos y barreras enfrentados por las personas usuarias de la Profilaxis Preexposición. **(4)** Evidencia la necesidad de estrategias orientadas a la información en salud.

## Introducción

A pesar de los avances científicos, diagnósticos y terapéuticos, la infección por el Virus de la Inmunodeficiencia Humana (VIH) sigue representando un desafío contemporáneo, y su impacto epidemiológico, social, cultural, económico y sanitario está ampliamente documentado en la literatura científica^(1)^. Considerando la magnitud del problema, el Consejo General de las Naciones Unidas propuso, en la Agenda para el Desarrollo Sostenible, el control de la epidemia para el año 2030, por medio de la información y la concienciación popular sobre las diferentes estrategias de prevención, como el uso de la Profilaxis Preexposición (PrEP)^(2)^.

La PrEP es una intervención biomédica altamente eficaz para la prevención del VIH, y constituye un componente esencial de la prevención combinada recomendada por la Organización Mundial de la Salud (OMS), implementada en varios países desde 2015^(3)^. En Brasil, fue incorporada al *Sistema Único de Saúde* (SUS) en 2017, estando dirigida a personas adultas y adolescentes sexualmente activas con mayor riesgo de infección^(4)^. Desde su implementación, 222.948 personas han adherido a esta estrategia. Sin embargo, a pesar del crecimiento en el número de usuarias y usuarios, la tasa de discontinuidad es elevada, lo que refleja los desafíos y barreras asociados al uso y la continuidad de la PrEP^(5)^.

Esta estrategia de prevención combinada implica una atención continua que incluye la concienciación sobre su uso entre las personas con riesgo sustancial de adquirir el VIH, la realización regular de pruebas, la captación, la adhesión y la retención en el cuidado^(6)^. No obstante, existen claras brechas en esta modalidad de atención, lo que sugiere la necesidad de nuevas investigaciones para comprender los determinantes, factores asociados y predictores de la adhesión, así como las barreras y desafíos en la implementación efectiva de la estrategia y en la carga desproporcionada de la infección evidenciada entre los grupos en situación de vulnerabilidad^(7-9)^.

De este modo, comprender los motivos para el uso de la PrEP, los beneficios percibidos y las posibles barreras enfrentadas por las personas expuestas al VIH es fundamental para el desarrollo de intervenciones eficaces que mejoren la atención brindada a esta población. En este sentido, el presente estudio tuvo como objetivo comprender las motivaciones, beneficios y barreras en el uso de la PrEP al VIH.

## Método

### Diseño del estudio

Se trata de un estudio descriptivo con enfoque cualitativo, conducido de acuerdo con las directrices propuestas por los *Consolidated Criteria for Reporting Qualitative Research* (COREQ)^(10)^ y fundamentado en el Modelo de Creencias en Salud (MCS)^(11)^.

### Referente teórico

El Modelo de Creencias en Salud (MCS) fue desarrollado en 1966 con el objetivo de explicar y comprender el comportamiento humano en el proceso de salud y en el control de enfermedades como el VIH^(12-13)^. Se trata de un modelo ampliamente referenciado y aplicado en investigaciones sobre comportamientos de salud y en el ámbito de la Enfermería. Según el MCS, para que una persona adopte una acción preventiva, debe creer que: a) es susceptible a una enfermedad o condición indeseada; b) su ocurrencia representa una amenaza importante para algún aspecto de su vida; c) llevar a cabo una intervención profiláctica es eficaz para reducir la susceptibilidad o la gravedad de la enfermedad (beneficios); y d) la acción preventiva no implica muchas barreras^(11)^.

Las creencias de una persona sobre estos factores determinan la probabilidad de que adopte comportamientos promotores de la salud y se utilizan para explicar la adopción de conductas que contribuyen a la prevención y al control de enfermedades, como el VIH, así como para identificar la aceptación de recomendaciones relacionadas con el cuidado de la salud^(12-13)^.

La susceptibilidad percibida se refiere a la percepción subjetiva del riesgo de contraer una determinada condición. La severidad percibida se refiere al juicio de la persona respecto a las implicaciones de un diagnóstico en su vida, como por ejemplo en el trabajo, la vida familiar o las relaciones sociales. Los beneficios comprenden las acciones positivas y las medidas preventivas relacionadas con las consecuencias del diagnóstico. Las barreras están asociadas a aspectos negativos, tales como obstáculos, molestias, costos financieros, entre otros^(11)^.

### Lugar de la recolección de datos

Los escenarios del estudio fueron dos Centros de Pruebas y Asesoramiento (CPA) de un municipio de gran tamaño del estado de São Paulo, Brasil. Cabe destacar que los CPA son unidades estratégicas para el asesoramiento y diagnóstico precoz de las Infecciones de Transmisión Sexual (ITS/VIH), y el acceso a estos servicios está condicionado a una cita previa y a una consulta con un equipo interdisciplinario para la realización de pruebas, orientación y asesoramiento.

Cabe mencionar que el municipio estudiado fue uno de los pioneros en el país en implementar la PrEP en su red de atención a la salud y, en total, cuenta con cinco CPA, los cuales están distribuidos en cinco distritos sanitarios. Por ello, se optó por seleccionar los dos CPA que fueron los primeros en ofrecer atención y dispensación de la PrEP en el municipio en cuestión: uno de ellos (CPA-A) está ubicado en una región de fácil acceso para la población que busca atención para el uso de la PrEP, y cuenta con personas en seguimiento por un período más prolongado; el otro (CPA-B) se encuentra en una región del municipio que atiende a personas que viven en zonas de mayor vulnerabilidad socioeconómica. Se buscó, por lo tanto, incluir a personas usuarias de la PrEP con diferentes características sociodemográficas.

### Período

El estudio se llevó a cabo entre los meses de febrero y mayo de 2023.

### Población y criterios de selección

En el municipio estudiado, la PrEP fue implementada en diciembre de 2017 y, hasta 2023, se registraron 1.623 personas usuarias de PrEP, de las cuales 1.199 recibieron al menos una dispensación en el SUS en los últimos 12 meses^(4)^. Participaron en el estudio personas seronegativas para el VIH, mayores de 18 años, que utilizaban la PrEP desde hacía al menos 90 días y que estaban en seguimiento en el servicio de salud. Los casos nuevos de PrEP fueron considerados criterio de exclusión, ya que se entiende que las posibles dificultades de adhesión son incipientes durante la fase inicial de la profilaxis. El muestreo se basó en el criterio de conveniencia intencional, es decir, se seleccionaron los participantes que asistieron en los días de recolección de datos y que cumplían con los criterios de inclusión establecidos.

### Participantes

Se invitó a dieciséis participantes a formar parte del estudio y no hubo desistencias ni rechazos, siendo 13 atendidos en el CPA-A y 3 en el CPA-B. La selección de los participantes tuvo como objetivo incluir hombres y mujeres en uso de PrEP de diferentes grupos etarios, con la finalidad de abarcar personas con distintas experiencias, percepciones y motivaciones.

### Recolección de datos

Los datos fueron recolectados mediante entrevistas individuales grabadas en audio, realizadas por la investigadora principal, quien fue capacitada para este estudio. El guion estuvo compuesto por preguntas abiertas basadas en el Modelo de Creencias en Salud (MCS), que abordaban aspectos relacionados con el uso de la PrEP. Se realizó un estudio piloto con cuatro participantes para analizar la viabilidad, adecuación y revisión del cuestionario elaborado, así como para facilitar la inserción de la investigadora en los escenarios y con los participantes del estudio. Cabe señalar que los participantes del piloto no fueron incluidos en el estudio, y el tiempo medio estimado de las entrevistas fue de 15 minutos.

Después del piloto, el guion con preguntas relacionadas con la experiencia de uso de la PrEP fue ajustado por las investigadoras del estudio en conjunto con el grupo de investigación con experiencia en la temática, y fue adaptado a su versión final, que contempló la caracterización de los participantes, así como las siguientes preguntas orientadoras: Susceptibilidad percibida: ¿Qué te motivó a buscar la PrEP?; Severidad percibida: ¿Por qué comenzaste a usar la PrEP? y ¿Qué factores influyeron en la adhesión a la PrEP?; Beneficios percibidos: Para ti, ¿cuáles son los beneficios de usar la PrEP?; Barreras percibidas: Para ti, ¿cuáles son las barreras/desventajas de usar la PrEP?. Para garantizar la confidencialidad de los datos, los participantes fueron identificados con la letra “P”, que designa “Participante”, junto del número correspondiente con su orden de inclusión (P1, P2 hasta P16).

La recolección de datos se realizó con la debida autorización de la Secretaría de Salud del municipio. La investigación fue aprobada por el Comité de Ética en Investigación (CEI), así como acordada con los responsables de los CPA. Se puso a disposición una sala privada y silenciosa para la realización de las entrevistas, que se llevaron a cabo semanalmente, en el horario de 8:00 a 17:00 horas, y fueron conducidas por la investigadora principal en los días en que las personas usuarias de la PrEP tenían citas programadas con los equipos de salud.

Los participantes que se encontraban en los CPA para su consulta de seguimiento relacionada con el uso de la PrEP y que, posiblemente, cumplían con los criterios de inclusión definidos en el estudio, fueron abordados individualmente por la investigadora principal, quien se presentaba y explicaba los objetivos del estudio en una sala privada, antes o después de la consulta médica. Tras la aceptación por escrito de los participantes, mediante la firma del Término de Consentimiento Libre e Informado (TCLI), la investigadora realizaba las entrevistas individuales, con el apoyo del guion y un grabador de voz.

### Análisis y tratamiento de los datos

Todas las entrevistas fueron transcritas en su totalidad y la recolección de datos se realizó hasta constatarse la saturación de la información, discutida entre los autores^(14)^. El análisis de los datos fue guiado por el MCS de Rosenstock^(11)^. Se llevó a cabo el Análisis de Contenido, según lo propuesto por Bardin^(15)^, considerando las siguientes etapas para la organización de los datos: preanálisis, exploración del material e interpretación, con tratamiento mediante codificación. La codificación y validación fue realizada por los propios autores, sin el uso de *softwares*, respetando el “árbol de códigos” de temas agrupados por significados temáticos para la posterior generación de categorías. Cabe destacar que las categorías fueron previamente definidas con base en el modelo teórico adoptado en el estudio.

### Aspectos éticos

El proyecto fue aprobado por la Secretaría de Salud del municipio estudiado y por el CEI de la institución responsable, bajo el dictamen n.º 4.852.588, del 16/07/2021. Todos los participantes fueron informados sobre los objetivos, beneficios y riesgos del estudio, con la posterior firma del TCLI.

## Resultados

La muestra estuvo compuesta por 16 participantes, con una edad promedio de 36,5 años. La mayoría era del sexo masculino, representando 15 (93,75%) participantes, de los cuales 13 (81,25%) eran Hombres que tienen Sexo con Hombres (HSH) y 10 (62,50%) eran solteros. En cuanto al tipo de pareja, cinco (31,25%) tenían una pareja fija, mientras que 10 (62,50%) mantenían parejas ocasionales. Con relación al nivel educativo, nueve (56,25%) tenían educación secundaria completa, tres (18,75%) tenían estudios de grado, uno (6,25%) tenía estudios de posgrado y otro (6,25%) había cursado formación técnica.

Las categorías resultantes del proceso de análisis respetaron las dimensiones del MCS y se expresaron en 22 temas. Al reflexionar sobre el uso de la PrEP para el VIH, los participantes reconocieron que, aunque existe tratamiento, no hay cura. Asimismo, identificaron los beneficios derivados del uso de la medicación y del seguimiento clínico. Sin embargo, también señalaron dificultades, relacionadas con el acceso a los servicios de salud, el estigma, los efectos secundarios y las incertidumbres sobre las repercusiones del medicamento. Las dimensiones del modelo propuesto, así como los temas que componen cada dominio, se detallan en la Figura 1.

**Figura 1-:** Temas emergentes según cada dimensión del Modelo de Creencias en Salud. Ribeirão Preto, SP, Brasil, 2023

*VIH = Virus de la Inmunodeficiencia Humana; ^†^ITS = Infecciones de Transmisión Sexual; ^‡^PrEP = Profilaxis Preexposición

### Susceptibilidad percibida

La percepción adecuada de las personas sobre su susceptibilidad a adquirir el VIH motivó la decisión de buscar la PrEP. Se identificaron diversos factores, como estar en una relación con una persona que vive con el VIH o desconocimiento/incertidumbre sobre la serología anti-VIH de la pareja sexual.

*Porque en el caso de ella* [la pareja]*, tiene el SIDA, tiene el VIH, no la enfermedad, solamente el virus, y ahí es una forma preventiva, como ella lo tiene, hace tratamiento, y yo tengo que hacerlo junto con ella.* (P1)

*Al principio de todo, lo conocí por la relación anterior que tenía, por el VIH de él, luego cuando quedé soltero seguí tomándolo porque me pareció más viable para protegerme, además del preservativo, y ahora con la nueva relación serodiscordante, lo que me motiva es la cuestión de la prevención misma, creo que es mejor tener ese cuidado adicional.* (P15)

*No fue solamente por esa relación, sino también por otras, nadie viene con un cartel en la frente que diga que tiene VIH y no todo el mundo tiene la libertad de hablar.* (P6)

Para los participantes, el uso de la PrEP proporciona mayor seguridad y libertad frente a la exposición y posible adquisición del VIH, de modo que su utilización también fue motivada por la percepción sobre las limitaciones del preservativo como método de prevención, debido a situaciones como accidentes con ruptura del mismo, o incluso por la resistencia y la dificultad para negociar su uso, o por preferir no utilizarlo con la pareja.

[...] *y no todo el mundo tiene la libertad de hablar, entonces si acaso ocurriera algún accidente, yo uso otros métodos, pero estaría más expuesto.* (P12)

*Bueno, en realidad, uno se siente un poco incómodo para hablar de esto, pero incluso en la relación con preservativo siempre quedaba, ¿cómo decirlo?, una desconfianza, un miedo, una sensación de que también podría haber contraído VIH, por ejemplo. Y también está la otra cuestión de que no me gusta la relación con preservativo, ¿entiendes?* (P8)

*Para sentirme más segura, para sentirme protegida, porque él* [la pareja] *no consigue tener relaciones con preservativo, y yo me sentía muy expuesta.* (P13)

La susceptibilidad para la adquisición del VIH también fue descrita en la incertidumbre dentro de las relaciones, incluso con parejas estables, debido a la falta de garantías sobre el comportamiento del otro. Asimismo, se mencionaron situaciones concretas como la adquisición de otras ITS o la infidelidad conyugal.

*Porque creo que es una protección adicional, en caso de que el preservativo se haya roto, y hasta pensé que si tuviera una pareja estable, nunca se sabe si uno va a ser engañado o no, entonces por lo menos del VIH sé que estaría bien.* (P12)

*Lo que me motivó fue que ya tuve una ITS, y la primera vez que me hice un examen entré en pánico. Entonces pensé que tenía que cuidarme, que necesito un seguimiento y cuidarme más.* (P14)

*Mi marido me engañó, entonces me di cuenta de que no se puede confiar en cualquier persona.* (P4)

La susceptibilidad percibida a la adquisición del VIH también fue identificada entre quienes mencionaron tener múltiples parejas sexuales o ejercer el trabajo sexual.

*Porque como no tengo una pareja fija, la exposición es muy grande y, por lo tanto, las probabilidades de contraer el virus también son muy altas. Por eso solicité el acceso a la PrEP para protegerme del virus.* (P7)

*Cuando uno entra en la prostitución, lo hace para obtener ganancias, ganar su dinero, mantenerse. Para mí es una profesión bastante buena, normal, porque a través de la prostitución conocí a mucha gente buena y conseguí mis cosas. Tenía mucho miedo de contraer la enfermedad y, además, en la prostitución hay precios para los servicios, ¿no? Y hay servicios que son mucho más lucrativos que otros, y yo no los ofrecía, así que siempre me quedaba atrás, muy limitada. Y luego, con la PrEP, lo logré.* (P15)

### Severidad percibida

Para los entrevistados, la infección por el VIH puede ocasionarles perjuicios a la salud, siendo una enfermedad que tiene tratamiento, pero es incurable. En contraste, se observa una percepción de menor riesgo o gravedad en caso de adquirir otras ITS.

*Entonces, fui más por ella, porque no sabía que existía prevención, sabía que existía tratamiento, ¿no?, para el VIH, y como ella tenía que tratarse, busqué dónde, y ahí vine a tratarme y como éramos pareja, me dijeron que tenía que hacer el tratamiento con ella, porque aún no hay cura, así que será mientras dure nuestra vida.* (P1)

*Hay otras* [refiriéndose a otras ITS]*, claro, todo vuelve a cero, ¿no? Sé que estoy protegido si se piensa en la hepatitis, que tiene vacunas y tal. Pero* [refiriéndose al VIH] *es mucho más grave, y me da mucho más miedo.* (P1)

### Beneficios percibidos

Los beneficios identificados con el uso de la PrEP fueron la sensación de mayor protección frente al VIH y la autonomía, lo que trajo como consecuencia sentimientos de mayor seguridad y bienestar psicológico. Sentirse seguro permitió a las personas disfrutar de su vida sexual de manera más placentera.

*Creo que es una liberación, una seguridad más, una oportunidad más en la vida.* (P5)

*La seguridad, ¿no? De la prevención combinada, de tener un medio más de prevención contra el VIH.* (P16)

[...] *el principal beneficio es esa cuestión de no tener la preocupación de la posibilidad de contagiarse* [...]. (P3)

*Tener un poco más de seguridad, ¿no? Porque los accidentes suceden, entonces usar la PrEP me deja un poco más confiado con relación al riesgo de contagiarme.* (P12)

*El beneficio también es que me siento con la mente más tranquila, no me quedo con esa preocupación de llegar aquí y encontrarme con un positivo. En ese sentido, es en lo que más me ayuda.* (P13)

*Ahí me sentí más seguro, ¿no? De tener una relación sin protección con mi pareja, incluso sabiendo que no tiene relaciones solamente conmigo. Me siento más seguro.* (P13)

Estar vinculado a un servicio de salud y poder realizar el seguimiento clínico ambulatorio regular fue señalado como un beneficio por las personas usuarias de la PrEP. La orientación del equipo de salud y el enfoque utilizado también desempeñaron un papel importante en la adhesión y el mantenimiento de la profilaxis. Estar en contacto con el servicio de salud y recibir información del equipo fue mencionado como un factor favorable para reflexionar sobre los hábitos y comportamientos que influyen en la situación de salud.

*Ahora creo que justamente por haberme sentido un poco más libre, eso me permitió reevaluar mis hábitos, mis comportamientos y, digamos, comenzar una vida diferente, de modo que hoy incluso la utilizo a demanda, porque realmente ya no tengo la vida que tenía antes, ¿no? Desde que empecé.* (P3)

*Cada tres meses tengo que venir, hacerme las pruebas y pasar con el médico. Tener este seguimiento médico a lo largo de la vida es el mayor beneficio para mí.* (P14)

### Barreras percibidas

Las barreras percibidas para el uso de la PrEP estuvieron relacionadas tanto con aspectos individuales, sociales y estructurales, como con la necesidad de consultas médicas regulares y la dificultad para conciliarlas con el trabajo, la dificultad para agendar dichas consultas, además de la complejidad del protocolo clínico, que requiere visitas de seguimiento y monitoreo para la realización de exámenes de laboratorio. También se mencionaron dificultades con la ingestión oral de los medicamentos, efectos secundarios e incertidumbres sobre los efectos del fármaco a largo plazo.

*Creo que lo que dificulta es la falta de cupos en las UBS* [Unidades Básicas de Salud]. *Los médicos tienen agenda, todo está organizado, pero a veces no conseguimos turno. Por ejemplo, intenté en algunas unidades cerca de casa y no había cupo.* (P11)

*Tomar una pastilla todos los días. Tratar de acordarse, por ejemplo, los hombres nunca necesitaron tomar anticonceptivos como las mujeres, entonces para ellos es más complicado tomarlo. Por eso quiero usar la inyección que va a salir.* (P12)

[...] *creo que ahora la doctora dijo que habrá una versión inyectable y será mucho mejor. Porque eso de la pastilla diaria es un fastidio.* (P15)

[...] *una desventaja es que es un medicamento nuevo, nadie sabe qué efectos tendrá de aquí a diez años.* (P9)

El estigma asociado al uso de la PrEP también fue mencionado como una barrera social, ya que esta estrategia aún es vista con tabú y prejuicio por parte de la comunidad.

*Existe ese estigma de que quien usa PrEP es más promiscuo, pero, en fin, no me importa esa opinión distorsionada.* (P5)

*[...] el hecho de que asocien la PrEP con la promiscuidad puede ser una desventaja, pero sería más un problema de la sociedad que del medicamento en sí.* (P14)

*Yo creo que es el juicio, ¿no? Porque parece que quien toma PrEP es promiscuo… a veces hasta lo es, pero ahí es donde sería interesante que la tomara. Creo que la comunidad LGBT pasa por una serie de cosas, y el hombre* [heterosexual] *tiene muchas ventajas.* [...] *entonces pienso que tachar a alguien de promiscuo puede acabar inhibiendo a las personas de usar la PrEP, incluso siendo una protección.* (P12)

## Discusión

Se comprenden las experiencias de las personas usuarias de la PrEP a la luz del MCS. Estos hallazgos revelan que la búsqueda por la PrEP fue motivada principalmente por la susceptibilidad percibida de adquisición del VIH, y que se configura como una estrategia de mayor seguridad entre las diversas adoptadas en la gestión del riesgo individual.

El perfil sociodemográfico de los participantes es similar al encontrado en otros países como España^(16)^, Australia^(17)^ y Bélgica^(18)^. Este hecho refuerza que las personas que buscan la PrEP tienen un mayor nivel educativo y, probablemente, un mayor acceso a información que influye en la toma de decisiones relacionadas con su salud^(19-20)^.

A pesar del alentador interés por esta nueva tecnología preventiva, la literatura señala que un menor nivel educativo, la estigmatización y el racismo están asociados a una menor intención de uso y búsqueda de la PrEP por parte de personas negras y mujeres^(21-22)^, lo que representa una preocupación, dado que en la población brasileña, las personas negras y con bajo nivel educativo son las que registran el mayor número de casos de SIDA^(23)^.

La serología del VIH de las parejas fue un aspecto relevante para el uso de la PrEP. Estar en una relación con una pareja que vive con el VIH fue identificado como una motivación para el uso de la PrEP. Este hallazgo refuerza la percepción de susceptibilidad percibida a la adquisición del VIH entre los participantes, además de destacar la PrEP como una estrategia eficaz para la prevención de la transmisión sexual del virus entre parejas serodiferentes. Las directrices nacionales^(24)^ e internacionales^(25)^ priorizan a estas parejas para la prevención primaria del VIH, incluyendo estrategias biomédicas como el Tratamiento como Prevención (TasP) y la PrEP.

Las parejas con serologías del VIH diferentes presentan mejores resultados en el tratamiento del VIH y mayor adherencia al uso de la PrEP^(26)^. Esto se debe a que las personas que viven con el VIH y están en seguimiento médico son incentivadas a invitar a sus parejas a buscar la PrEP y a apoyarse mutuamente para recordar tomar las pastillas^(9)^. Además, la PrEP actúa frecuentemente como un puente hacia la Terapia Antirretroviral (TAR) entre las parejas serodiferentes. Desde esta perspectiva, la pareja seronegativa hace uso de la PrEP hasta que la pareja seropositiva complete seis meses de uso regular de TAR y alcance niveles indetectables de la carga viral plasmática^(27)^.

No obstante, el desconocimiento o la incertidumbre sobre la serología del VIH entre parejas ocasionales también se configuró como un motivo para buscar la profilaxis en estos hallazgos. Las personas que viven con el VIH siguen considerando que revelar su serología es una práctica arriesgada y conviven con el diagnóstico en secreto, por miedo a la repercusión que esta información podría tener en la comunidad^(28-29)^.

La susceptibilidad percibida al VIH también se refleja en la posibilidad de fallos o dificultades en el uso del preservativo. Aunque el preservativo es un método preventivo accesible, de bajo costo y sin efectos secundarios, su efectividad puede verse comprometida por fallos como la rotura o el deslizamiento. Además, el uso constante del preservativo representa un desafío para muchas personas, con baja adherencia en la población brasileña, incluidas aquellas con mayor vulnerabilidad al VIH, como los jóvenes, los HSH y las trabajadoras y trabajadores sexuales^(30-31)^. Un estudio brasileño identificó que el 85% de los HSH informaron un uso inconsistente del preservativo, y que la probabilidad de uso inconsistente aumentaba entre aquellos que tenían parejas estables, que practicaban sexo anal en posición insertiva o que ya habían tenido una ITS en el pasado^(32)^. Estos datos subrayan la necesidad de intensificar la concienciación sobre la importancia del preservativo en la prevención de otras ITS, especialmente entre estas poblaciones.

En este contexto, la PrEP fue mencionada como un método viable para quienes no utilizan el preservativo de forma constante, y también fue considerada una estrategia más eficaz, que proporciona mayor seguridad y bienestar psicológico a sus usuarios. Estos hallazgos son coherentes con estudios internacionales que demuestran que la reducción de la preocupación por el riesgo de transmisión durante el acto sexual está directamente relacionada con la mejora de la calidad y satisfacción sexual entre las parejas^(33-35)^. Además, a medida que surgen nuevas estrategias de prevención y se vuelven más populares, se espera que la percepción del riesgo de adquisición del VIH no se base exclusivamente en el uso del preservativo^(34)^.

Las personas que se perciben en riesgo de exposición al VIH debido a la incertidumbre en las relaciones, incluso en las parejas estables, mencionaron la PrEP como una forma de garantizar el autocuidado. Esta percepción surge de la falta de certeza sobre el comportamiento de la pareja, especialmente tras experiencias previas de adquisición de otras ITS o de infidelidad conyugal. La literatura señala que las parejas en relaciones ocasionales enfrentan mayores desafíos en la adopción de estrategias de prevención, debido a la falta de intimidad para discutir dichas medidas y al hecho de que muchos encuentros ocurren de manera no planificada^(36-37)^.

Son dignos de atención los hallazgos de este estudio, que indican la búsqueda de la PrEP tras el diagnóstico de una ITS, al relacionarse con una pareja con serología positiva para el VIH o por mantener múltiples parejas, además de manifestar inseguridad en utilizar únicamente el preservativo como estrategia de prevención. Estos resultados sugieren que las campañas de concienciación sobre los factores de riesgo para el VIH y sobre la prevención combinada están generando un impacto positivo en la población. La percepción de riesgo de estas personas refleja factores que, de hecho, han sido destacados por autoridades sanitarias y por investigadores del área^(38)^.

Los hallazgos revelaron la autopercepción de riesgo de los participantes con respecto a diversas otras posibles ITS. Esta percepción puede estar relacionada con el hecho de que dichas infecciones no son consideradas por las personas como problemas de salud tan graves como el VIH. En consecuencia, la información, la motivación y las competencias necesarias para movilizar acciones de prevención frente a estas infecciones pueden ser abordadas de manera diferenciada^(39)^.

La construcción social del VIH ha evolucionado a lo largo del tiempo, incorporando distintos discursos y significados. En los inicios de la epidemia, el VIH fue considerado un “cáncer gay”, percibido como una sentencia de muerte, y continuó rodeado de connotaciones negativas a lo largo de los años^(40)^. Desde entonces, los avances en el ámbito científico han permitido que el VIH sea manejado como una condición crónica, lo que ha resultado en una mayor sobrevida y calidad de vida para las personas que viven con el virus. Sin embargo, el VIH sigue siendo una infección con repercusiones complejas para la salud, especialmente en situaciones de falla terapéutica^(40-41)^_._

Algunos estudios han demostrado la alta efectividad de la PrEP como estrategia de prevención del VIH^(35,37,42)^ y, por ello, su uso continuo ha tenido un impacto positivo en la experiencia de las actividades sexuales. La PrEP no solamente proporciona protección, sino que también reduce la amenaza para la relación, aliviando el miedo y permitiendo la preservación de los vínculos afectivo-sexuales^(43)^.

Además, independientemente de la serología frente al VIH de las parejas sexuales, la PrEP se presenta como una alternativa viable para aquellas personas que no se adaptan al uso regular del preservativo, como ha sido evidenciado en otros estudios^(34-35)^. Adicionalmente, el uso de la PrEP mejora la autoeficacia, haciendo que las personas usuarias se sientan más responsables y en control de sus decisiones sexuales, sin depender de la honestidad de sus parejas respecto a su estado serológico^(35)^.

Otro punto destacado fue el seguimiento clínico asociado al uso de la PrEP, considerado una oportunidad para la promoción de la salud, con pruebas frecuentes, evaluación continua y solicitud regular de exámenes. El contacto constante con el equipo de salud fue señalado como un aspecto positivo, ya que permite reflexionar sobre los hábitos y mejorar el autocuidado entre los participantes. Estos hallazgos coinciden con estudios internacionales que muestran que las personas usuarias de PrEP reconocen el paquete de atención ofrecido junto con esta tecnología preventiva como una ventaja para la salud^(35,39)^.

El Ministerio de Salud de Brasil^(24)^ recomienda un conjunto de intervenciones asociadas al seguimiento clínico de personas usuarias de PrEP, incluyendo evaluaciones generales, pruebas de detección del VIH, identificación de otras ITS como clamidia, sífilis, gonorrea y hepatitis virales, vacunación contra el Virus del Papiloma Humano (VPH), así como monitoreo de la función renal y prueba de embarazo. De este modo, los beneficios del seguimiento clínico de la PrEP van más allá de la prevención del VIH, ya que permiten el diagnóstico precoz de otras infecciones, el acceso a diversas estrategias preventivas, evaluaciones periódicas de salud, además de constituir una puerta de entrada al SUS.

Además del acompañamiento en salud, la construcción de una relación interpersonal positiva con los profesionales del equipo fue un factor importante para la adherencia a la profilaxis. Esto refuerza que un entorno acogedor, con una atención libre de estigmas y prejuicios, favorece el vínculo de las personas usuarias de esta tecnología preventiva con el servicio de salud, fortaleciendo así la adherencia al seguimiento clínico^(44)^.

Las barreras para el uso de la PrEP abarcan aspectos individuales, sociales y estructurales, como se describe en un estudio de revisión de la literatura^(9)^. Entre las barreras estructurales, se destacan la complejidad del protocolo clínico, que requiere visitas regulares de seguimiento y monitoreo, incluyendo la realización de pruebas de laboratorio, así como el tiempo prolongado de espera para las consultas médicas. Estos desafíos también han sido identificados en otros estudios como obstáculos al uso continuado de la PrEP^(45)^.

La adherencia diaria a los medicamentos, especialmente entre los jóvenes y los hombres que no tienen el hábito de realizar un seguimiento preventivo regular y a largo plazo, las incertidumbres sobre los efectos secundarios de la PrEP a largo plazo y la necesidad de visitas frecuentes para el seguimiento clínico fueron identificadas como barreras individuales para la continuidad del uso de la PrEP. Hallazgos similares se han reportado en otros países, donde los participantes señalaron la dificultad de recordar tomar las pastillas como un desafío común entre las personas usuarias de PrEP^(37)^.

Tras el inicio de la PrEP, la primera dispensación será de 30 días. Las dispensaciones siguientes se realizarán cada 90 días o, dependiendo de la adherencia al medicamento, cada 120 días, según la evaluación del profesional de salud. Si la persona usuaria no recibe la cantidad total de PrEP, podrá obtener el resto sin necesidad de una nueva prueba de VIH, siempre que el formulario esté dentro del período de validez. Las personas con adherencia continua durante al menos un año podrán tener el seguimiento clínico ampliado a seis meses, con ajustes en la dispensación según la disponibilidad local. En caso de presentar síntomas de ITS, deberán regresar al servicio de salud^(24)^.

Los regímenes antirretrovirales orales son altamente eficaces para la prevención del VIH, presentan toxicidad mínima y ofrecen la conveniencia de una dosificación sencilla, con un solo comprimido diario. No obstante, para abordar los desafíos relacionados con la adherencia, se han estudiado alternativas de acción prolongada, como los antirretrovirales inyectables, los implantes subcutáneos de larga duración y los anillos vaginales de liberación controlada. Estas opciones representan estrategias prometedoras, al ofrecer dosis menos frecuentes, como agentes orales de administración semanal o terapias de acción prolongada por vía parenteral. Tales enfoques pueden resultar especialmente útiles en contextos donde el uso diario de medicamentos orales es complicado o la adherencia al tratamiento es insuficiente^(46)^.

Aunque muchos participantes señalaron el seguimiento presencial como un desafío debido a sus horarios laborales, han surgido nuevas estrategias de acceso en el ámbito de la PrEP para superar esta barrera. Entre estas iniciativas se incluyen las consultas en línea, la entrega domiciliaria de los comprimidos, la flexibilización de los puntos de retiro de la medicación y la incorporación de otras categorías profesionales como prescriptores de PrEP. Estas medidas han facilitado el acceso a la profilaxis y han contribuido a mejorar la adherencia y la regularidad en el seguimiento clínico^(24)^.

Existen aún oportunidades para mejorar la adherencia a la PrEP mediante la implementación de intervenciones individuales personalizadas y modelos que amplíen el acceso, especialmente en contextos no tradicionales^(44)^. Una revisión de la literatura reveló que los modelos de prestación de servicios están predominantemente concentrados en clínicas y hospitales especializados en el tratamiento de ITS, VIH y clínicas de salud sexual, particularmente en países de altos ingresos^(47)^.

En diversos países, los enfermeros desempeñan un papel de liderazgo en los servicios de PrEP^(48)^, siendo fundamentales para ampliar el acceso a la atención primaria, especialmente entre poblaciones en situación de vulnerabilidad^(49)^. Su actuación promueve la alfabetización en salud sobre la PrEP al VIH, al educar y monitorear a las personas, gestionar servicios y, en consecuencia, ampliar el acceso y la adherencia a esta estrategia preventiva^(50)^. Modelos de atención liderados por enfermeros, como la clínica PrEP-RN^(51-52)^, han demostrado que la relación de confianza y el apoyo brindado a las personas usuarias de PrEP contribuyen significativamente a la adherencia. Por lo tanto, repensar el modelo de prestación de servicios en Brasil, fortaleciendo el rol de otros profesionales de salud capacitados, puede reducir las barreras de acceso y ampliar el alcance de la PrEP.

Los modelos orientados a la autogestión han demostrado resultados positivos, incluyendo el uso de autotests para el VIH y pruebas para otras ITS, lo que permite a las personas sustituir las visitas presenciales a clínicas o laboratorios por prácticas de autocuidado en el hogar^(47)^. Además, las intervenciones de salud móvil (*mHealth*) y salud digital han mostrado ser prometedoras en la prevención del VIH, especialmente mediante aplicaciones para teléfonos inteligentes. Estas tecnologías, cuando se integran a los servicios de PrEP y se asocian con pruebas accesibles, ayudan a superar diversas barreras. Tienen el potencial de alcanzar e involucrar de forma anónima a poblaciones que frecuentemente quedan al margen de los esfuerzos tradicionales de prevención, además de ofrecer la entrega rápida y en tiempo real de programas, con costos de implementación relativamente bajos^(49)^.

El conocimiento sobre los efectos secundarios y las incertidumbres en cuanto a los impactos del medicamento a largo plazo representan barreras para las personas usuarias de la PrEP. Aunque los beneficios son ampliamente reconocidos, los posibles efectos adversos de los antirretrovirales están bien documentados, con base en la experiencia acumulada de su uso en el tratamiento del VIH. No obstante, una revisión sistemática sugiere que los riesgos de eventos adversos relacionados con toxicidad renal varían según la edad y la depuración basal de creatinina en personas usuarias de PrEP. En este contexto, la realización de un tamizaje menos frecuente o incluso opcional para personas jóvenes sin comorbilidades renales podría reducir las barreras para la implementación y el uso continuo de la PrEP^(52)^.

Este estudio puede contribuir a orientar prácticas de cuidado centradas en la persona, favoreciendo la adherencia al tratamiento a partir de la comprensión de sus demandas y la oferta de orientaciones e instrucciones adecuadas. Además, refuerza la importancia de desarrollar campañas, en colaboración con el Ministerio de Salud, dirigidas a distintos públicos, con el objetivo de promover la PrEP como una estrategia eficaz de prevención y combate al VIH.

Los hallazgos de este estudio resaltan el papel central de la Enfermería en la reducción de las desigualdades en salud^(53)^, especialmente en lo que respecta al acceso a la PrEP. El análisis de las experiencias de las personas usuarias de PrEP evidencia la relevancia de la percepción del riesgo y del soporte continuo para garantizar la adherencia a esta tecnología preventiva. Los resultados destacan la necesidad de invertir en la práctica cotidiana de los profesionales de Enfermería, con foco en poblaciones vulnerabilizadas, reforzando su capacidad de adaptar intervenciones socioculturales y liderar iniciativas orientadas a la equidad en salud. Se recomienda fortalecer la formación y el liderazgo de los enfermeros, promoviendo el uso de tecnologías digitales y modelos de atención descentralizados, alineados con las demandas sociales.

Estos hallazgos deben interpretarse a la luz de algunas limitaciones. En primer lugar, la muestra fue homogénea, compuesta predominantemente por hombres cisgénero, blancos y que tienen relaciones sexuales con otros hombres. Además, los datos fueron recolectados en un municipio de gran tamaño en el interior del estado de São Paulo, que presenta diferencias socioculturales y económicas en comparación con otras regiones de Brasil.

## Conclusión

El estudio permitió comprender las decisiones, percepciones, beneficios y barreras para la adhesión y continuidad del uso de la PrEP, desde la perspectiva de las personas que la utilizan. El uso de la PrEP fue motivado por la percepción de susceptibilidad, la serodiferencia y el desconocimiento/incertidumbre sobre la serología anti-VIH de la pareja sexual. Además, la gravedad atribuida a la infección por el VIH y su asociación con una enfermedad incurable influyeron en las decisiones relacionadas con la salud. Entre los beneficios señalados se destacan la protección frente al VIH, la seguridad y el bienestar psicológico, así como la posibilidad de establecer un vínculo con un servicio de salud y realizar un seguimiento clínico-ambulatorio de forma regular.

En cuanto a las barreras, estas reflejaron aspectos individuales, sociales y estructurales, como la dificultad de acceso y programación de las consultas, vinculadas a la complejidad del protocolo clínico que requiere visitas de seguimiento y monitoreo para la realización de exámenes de laboratorio, además de la dificultad para ingerir los medicamentos por vía oral, efectos secundarios e incertidumbres sobre los efectos del medicamento a largo plazo. Repensar el modelo de prestación de servicios de PrEP para la prevención del VIH debe incluir la ampliación del rol del profesional de enfermería en la atención a las personas usuarias. Es esencial que el abordaje sea integral, considerando tanto las barreras individuales como las estructurales, con el fin de fortalecer la efectividad de la estrategia y avanzar en el control de la epidemia de VIH hacia 2030.

## Data Availability

Los conjuntos de datos relacionados con este artículo estarán disponibles previa solicitud al autor correspondiente.
